# CO _2_ sequestration potential in Depleted Hydrocarbon fields – A geochemical approach

**DOI:** 10.12688/openreseurope.19280.2

**Published:** 2025-04-04

**Authors:** Eleni Gianni, Pavlos Tyrologou, Dounya Behnous, Márton Pál Farkas, Paula Fernández-Canteli Álvarez, Jesús García Crespo, Ricardo Chacartegui Ramirez, Nikolaos Koukouzas, Júlio Carneiro

**Affiliations:** 1 Centre for Research and Technology Hellas (CERTH), Egialias 52, Marousi, 151 25, Greece; 2 Department of Environment, Ionian University, Zakynthos, M. Minotou-Giannopoulou 26, 29100, Greece; 3 Research Projects & Development Unit European Federation of Geologists, Brussels, Belgium; 4 Parque do Alentejo de Ciência e Tecnologia, Converge!, Évora, 7005-841, Portugal; 5 Geoenergy, GFZ German Research Centre for Geosciences, Telegrafenberg, Potsdam,, 14473, Germany; 6 Instituto Geológico y Minero de España (IGME-CSIC), Madrid, Ríos Rosas, 23, 28003, Spain; 7 Department Ingeniería Energética, Universidad de Sevilla, Sevilla, Camino de los Descubrimientos s/n, 41092, Spain; 8 Laboratory of Engineering for Energy and Environmental Sustainability, Universidad de Sevilla, Seville, 41092, Spain; 9 ICT/IIFA, Geosciences Department, Universidade de Évora, Évora, R. Romão Ramalho 59, 7000-671, Portugal

**Keywords:** CO2 storage; depleted hydrocarbon fields; energy storage; geochemical interactions; underground storage

## Abstract

**Background:**

The CO _2_ emissions reduction is crucial for the energy transition. New technologies for CO _2_ capture and storage are under development, such as CEEGS ^1, 2^. Porous media and rock caverns are geological formations of high interest for such technology. Among them, depleted hydrocarbon fields (DHF) gain ground due to existing reservoir knowledge and already established infrastructure which decreases the cost. However, one of the major problems caused during CO _2_ storage in DHF is the interactions between the injected CO _2_ and the remaining fluids.

**Methods:**

In this study, the potential CO _2_ storage in DHF was investigated. Marismas 3 was used as a hypothetical model area for the examination of CO _2_ interactions with a carbonate-silisiclastic reservoir. PHREEQC software ^1^ was used to investigate reservoir rock/water/remained gas (CH _4_) interactions followed by interactions taking place after the CO _2_ injection. Different scenarios were used for the CO _2_ concentration and behaviour in the reservoir. To make the system more complex and generic, the CMG-GEM software ^3^ was utilized to examine the long-term sequestration of CO _2_ through dissolution trapping, residual trapping, and lateral migration in a reservoir analogue to the Marismas field, but at higher depth, compatible with the CEEGS technology.

**Results:**

During the CO _2_ injection, carbonic acid was formed, causing a dissolution of several minerals, leading to siderite and clay minerals precipitation, which may cause problems to the permeability of the system. The colloidal nature of siderite and the Ca-montmorillonite swelling properties are of high concern for pore throat clogging. The other newly formed mineralogical phases are not threatening the reservoir quality. CMG-GEM validated the critical phase of CO _2_ plume establishment.

**Conclusions:**

The proposed DHF is promising for real-world underground applications fitting to CEEGS technology as the newly formed minerals that could cause failures can be easily controlled by anthropogenic changes in the reservoir parameters.

## Plain Language summary

**Background:**

The CO _2_ emissions in the atmosphere enhance global warming. The European Union set the lines for the energy transition. New technologies dealing with CO _2_ capture and storage are under development, such as CEEGS ^1^. For underground CO _2_ storage, two main geological categories are required; the geological formations with high porosity (porous media), and the cavities developed in hard rocks. Each category is subdivided into further clasifications with different technological requirements, influencing the required capital cost. The most economically attractive option is the depleted hydrocarbon fields (DHF) as there is an existing experience of reservoir characterization and already established infrastructure which critically decreases the cost. However, the geochemical interactions between the injected CO _2_ and the reservoir’s remaining fluids may cause problems.

**Methods:**

In this study, the potential CO _2_ storage in DHF was investigated. Marismas 3 gas field was used as a hypothetical model area for the examination of CO _2_ interactions with a carbonate-silisiclastic reservoir.

PHREEQC software ^1^ was used to investigate the reservoir rock/remained water/remained gas (CH _4_) interactions followed by the interactions taking place after the CO _2_ injection. Several scenarios were examined depending on the CO _2_ concentration and behaviour in the reservoir. To make the system more complex and generic, the CMG-GEM software ^3^ was utilized combining dynamic fluid flow and geochemistry, examining the long-term sequestration of CO _2_ through dissolution trapping, residual trapping, and lateral migration.

**Results:**

During the CO _2_ injection, carbonic acid was formed, causing a dissolution of several minerals and siderite and clay minerals’ precipitation. The colloidal nature of siderite and the swelling properties of Ca-montmorillonite are of high concern for pore throats clogging. However, the other newly formed mineralogical phases are not threatening the reservoir quality. CMG-GEM validated the critical phase of CO _2_ plume establishment.

**Conclusions:**

The proposed DHF is promising for real-world underground applications fitting to CEEGS technology.

## Introduction

Climate change is a global phenomenon strongly correlated with water, food, and energy changes. Thus, the energy-climate nexus is a key challenge for the sustainable development achievement ^4^. Due to the increase of industrialisation, an increase in the released CO _2_ emissions in the atmosphere was observed, enhancing the global warming phenomenon as the main component of greenhouse gasses ^5^. However, the continuous population development and economic growth require higher energy demands. Comparing with previous decades, this demand is slower increasing with an average of 0.7 % per year through 2050 than a percentage higher than 2 % average from 2000 to 2015 ^6^. Thus, the European strategies aim for the energy policies’ rapid changes to achieve efficient energy demands combined with greenhouse gas reduction. The 2015 Paris Agreement proposed the maintenance of earth’s temperature at 1.5–2.0 °C and the mitigation of climate change consequences ^7^. Moreover, the EU Energy Roadmap 2050 planed a reduction of greenhouse gas by 80–95 % by 2050 compared to the 1990 base. Hence, proper management of CO _2_ emissions aiming to the reduction of the quantities in the atmosphere and their use for energetical purposes is crucial for the sequence of the lines of the EU.

To achieve the CO _2_ emissions reduction, the CO _2_ capture and storage (CCS) technologies were developed. Two main categories were occurred; the storage in above ground facilities and in underground geological environments. The underground facilities are more favourable as they can store a higher amount of CO _2_, have a limited footprint as require lower land use for the aboveground installations, they are significantly less influenced to external factors and they are more safe due to the tightness of the caverns as well as the considerable distance from the biosphere and hydrosphere ^8^. The CO _2_ underground storage was developed after understanding the different geological formations potential to contain tremendous amounts of hydrocarbons for million of years, which enhanced by the remarkable tightness and rock integrity of such formations. Potential geological environments for the carbon storage are the porous media and the hard rock caverns. The porous media are subdivided in depleted hydrocarbon reservoirs and in saline aquifers. The hard rock caverns separated in salt caverns and lined or unlined rock caverns ^9^.

CO _2_ based electrothermal energy and geological storage system, known as CEEGS ^2^, is one of novel technologies aiming to combine renewable energy storage systems, the transcritical CO _2_ cycle, the geothermal heat extraction and partial CO _2_ storage in geological formations. The concept is to integrate the transcritical CO _2_ cycles with underground energy storage through simultaneous CO _2_ geological storage and geothermal heat extraction. By this way, the advantages of Carbon Capture, Utilisation and Storage (CCUS) and renewable energy storage technologies with respect to efficiency and profit will be enhanced with a simultaneous minimal environmental impact.

Depleted hydrocarbon reservoirs are of high interest for CO _2_ storage for both scientific and industrial community compared to the other categories in terms of CO _2_ storage capacity, experience of reservoir characterisation, already established oil or gas well infrastructure, sealing performance as well as storage operability ^10– 14^. The CO _2_ storage in depleted hydrocarbon fields supposed to be one of the most realistic ways to reduce carbon emissions while it is the most economical-affordable method ^15^. The known globally storage capacity of the available depleted hydrocarbon fields (DHF) is estimated to be close to 390–750 Gigatons which is approximately ten times the current annual CO _2_ emissions worldwide ^13^. Despite the advantages of DHF, critical disadvantages influence the efficiency of CO _2_ storage and must be addressed prior its injection. More specifically, the problems can be summarised in; i. problems related to the evaluation method of storage potential and its applicability, ii. potential leakage of CO _2_ and description of its mechanism, and iii. the interactions between the injected CO _2_ and the remaining fluids ^10^.

The purpose of this study is to investigate the potential interactions between the injected CO _2_ in a model DHF by a geochemical computer simulation method. As a model DHF, the depleted gas field of the Marismas fields in South Spain was selected, supposing that represents a range of depleted gas reservoirs with host rocks of carbonate-siliciclastic nature. Firstly, the existing geochemical interactions taken place in the Marismas field were specified, describing the host rock, remaining fluids (i.e. CH _4_), and remaining water interactions. Secondly, the interactions of the complex Marismas field system and the injected CO _2_ were examined. Moreover, the thermal-hydraulic-chemical simulation of the depleted gas reservoir using the CMG-GEM compositional simulator ^3^, based on the features of the Marismas, was examined. The primary objective was to assess the technical feasibility of implementing a depleted hydrocarbons reservoir for the CEEGS technology. The models designed based on the needs of CEEGS concept, requiring a supercritical state of the injected CO _2_, while the results of the study may be helpful for other CO _2_ storage technologies too. The study represents a first attempt for Marismas field area conceptualization based on data from traditional geological mapping and primary field data, considering the selected DHF as a primary model of a carbonate-siliciclastic reservoir pending further research and data availability.

## Methods

### Study area

Among the suggested reservoir types for CEEGS technology implementation, the depleted reservoir (gas or oil) holds notable importance. These reservoirs are discarded remnants from the oil and gas industry. Rather than being left abandoned, they can be repurposed for specific applications, particularly within the realm of CEEGS technologies. The peculiarity of many of these reservoirs lies in the remnanent low pressure, which ensures optimal functioning of CEEGS technology. It is crucial that the depleted reservoir pressure does not fall below this critical minimum pressure. This ensures that the injected CO _2_ can be back-produced to the surface when needed (without requiring energy input that would decrease the efficiency of the system, and reach the wellhead under supercritical conditions.

An additional challenge is posed by the Joule-Thomson effect on the injection well. Low pressures at the reservoir can result in a strong temperature decrease of the CO _2_ as it expands at the bottomhole. This process can lead to damage to materials of the wells or to clogging of the system due to the formation of CO _2_ hydrates ^16, 17^.

The objective of the numerical model of the depleted hydrocarbon field is to assess the feasibility of this scenario to the CEEGS system from a technical point of view. 

For the investigation of geochemical reactions during the CO _2_ injection into a depleted hydrocarbon reservoir, the Marismas fields were selected as a mock-up target area. Although it is not claimed to store CO _2_ for energy storage purposes at the Marismas field, some of its characteristics are fairly representative of DHFs where such could be considered. In this study we utilized only data in the public domain, and whenever there was a complete absence of information, expert-based assumptions were made regarded as realistic for the intended aims of the study.

**Figure 1.**
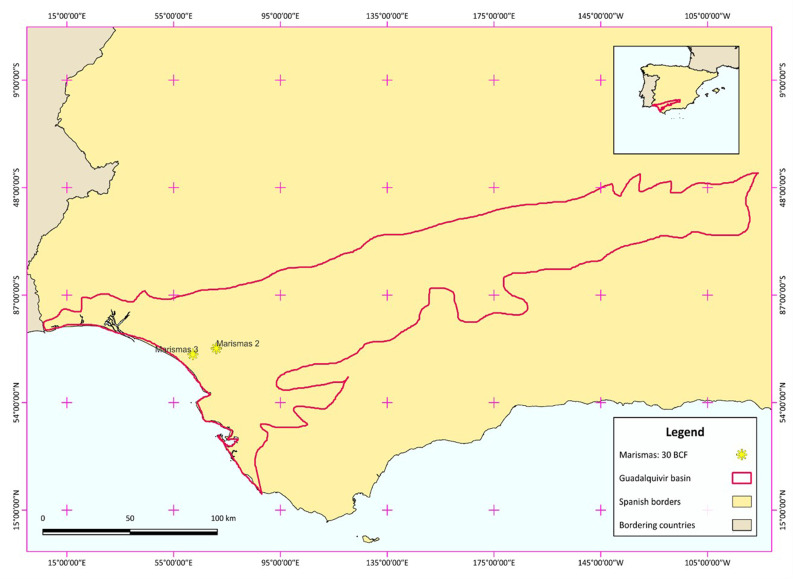
Study area of Marismas fields, showing the Marismas 3 field which is the selected model of depleted hydrocarbon field.

The Marismas fields are operational gas fields belonging to Guadalquivir-Cadiz Miocene Foreland Basin in the South Spain, as given in the Figure 1 ^18^. They are characterised by high porosity and permeability present in the onshore Guadalquivir and offshore Gadiz Gulf sandy turbiditic formations. The hydrocarbon traps of the area are mostly stratigraphic and the gas generation origin is mainly biogenic, produced by marine sediments and especially clays or shales which are located some meters under the reservoirs.

The Guadalquivir basin generated during Miocene due to the collision between the Iberian and African plates. The basin’s depth is shallow, ranging from 500 m depth in the east basement deepening to 1200 m depth to the west. The area divided in four main stratigraphic sequences ^19^: 1. The basal transgressive calcarenite which is the base of the basin, 2. The Middle and 3. Late Miocene Guadalquivir formation consisted of shallow marine sand units continuing into deep-water turbidites, and 4. The Pliocene formation which overlies unconformably the Miocene unit. The lithology of the basin differentiates based on the geographic location. Marismas fields occur between Huelva and Sevilla provinces with thin sediments composed of calcarenite and siliciclastic deposits ^19^.

The calcarenite composing the reservoir is a mixture of calcite (70 %), iron-rich dolomite (25 %), quartz (5 %) and clay minerals traces with a dominance of illite ^20^. Neogene deposits of the lower-northwestern margin of Guadalquivir Basin are divided into four lithostratigraphic units ^21^: 1. mixed carbonate–siliciclastic deposits of the Niebla Formation (bottom) ^22, 23^, 2. greenish–bluish clays of the Arcillas de Gibraleón Formation ^23^, 3. fossiliferous sands and silts of the Arenas de Huelva Formation ^23^, and 4. sands of the Arenas de Bonares Formation ^24^.

As the specific mineralogical composition of the present area is not specified in the literature and the Marismas fields were used as a model depleted hydrocarbon field for the understanding of hydrogen interactions in similar environments, several assumptions were used for the specification of the main mineralogy of the reservoirs. The estimation of main mineralogical phases was performed taking into consideration the semiquantitative mineralogical analysis of the Gulf of Cadiz area, as it was specified by 25. Quartz, alkali-feldspars, illite-mica, kaolinite and Fe-oxides are reported in the area. However, as marine sand units form the Guadalquivir basin and the exact mineralogy of carbonate-siliciclastic deposits of the Niebla is not known, the percentage of carbonates supposed to be slightly higher than quartz. The Gibraleón Formation mainly consists by marls and clays with benthic remains and plankton ^22^. At the base of both Gibraleón and Huelva Sands Formations, there are glauconite-rich horizons, indicating a paleoenvironment with shallow depths at the sediment-water interface. Amounts between 5 to 21 % of glauconite were examined in the areas. The glauconite-rich horizons, except of glauconite, contain also quartz, alkali feldspars and clay minerals as main mineralogical phases, while impurities of ilmenite, hematite, rutile, jarosite, and Fe-rich minerals were also present in the areas ^26^. From the group of clay minerals, illite and kaolinite prevailed in the area, while smectite with its characteristic swelling properties, occurred only as an impurity. The Huelva Formation is rich in quartz, feldspars, phyllosilicates (clay minerals, and muscovite), illite, and chlorite. Mixed-layer clay minerals with the characteristic example of illite-muscovite are also present in the area ^27^. The heavy fraction of sediments samples of the area verified the presence of magnetite, ilmenite, pyrite, hematite, limonite, goethite, andalusite, hornblende, epidote, sillimanite, kyanite, tourmaline, zircon, rutile, apatite, titanite and in smaller appearance granate, and galena. The heavy fraction covers a percentage of 5 to 15 % of the total samples and for this reason, the average of these percentages was assumed, which means the 10 %. Concerning the detection limits of the analytical techniques used for the identification of the minerals that are almost a 3 % of the sample, the minimum concentration of the above minerals specified to 0.3 %. The estimated mineralogical phases of the area are given in the Table 1. Estimated main mineralogical phases and impurities of the studied area with the empirical formula and the database used for the PHREEQC calculations., keeping into consideration the aforementioned knowledge of the percentages, mainly used for the mineralogical impurities. Some of them assumed to be present in higher concentrations related to their abundant presence or in lower due to the lack of their extend ^27^.

**Table 1.** Estimated main mineralogical phases and impurities of the studied area with the empirical formula and the database used for the PHREEQC calculations.

Minerals	Main	Impurities	Empirical formula	Estimated quantities %	Database
Calcite or/and Aragonite	+		CaCO _3_	26	phreeqc.dat
Dolomite	+		CaMg(CO _3_) _2_	9.2	phreeqc.dat
Quartz	+		SiO _2_	26	phreeqc.dat
Alkali Feldspars	+		KAlSi _3_O _8_	1.6	phreeqc.dat
Muscovite (K-mica)	+		Kal _3_Si _3_O _10_(OH) _2_	1.8	phreeqc.dat
Illite	+		K _0.6_Mg _0.25_Al _2.3_Si _3.5_^o^_10_(OH) _2_	3	phreeqc.dat
Kaolinite	+	+	Al _2_Si _2_O _5_(OH) _4_	8.5	phreeqc.dat
Chlorite	+		Mg _5_Al _2_Si _3_O _10_(OH) _8_	2	phreeqc.dat
Glauconite	+		K _0.75_Mg _0.25_Fe _1.5_Al _0.50_Si _3.75_^o^_10_(OH) _2_	13	sit.dat
Magnetite		+	Fe _3_O _4_	0.6	
Ilmenite		+	Fe ^2+^TiO _3_	1.0	
Hematite		+	Fe _2_O _3_	1.0	
Goethite / Limonite		+	FeOOH	0.6	
Pyrite		+	FeS _2_	0.4	
Hornblende		+	Ca _2_(Fe ^2+^_4_Al)(Si _7_Al)O _22_(OH) _2_	0.4	
Tourmaline		+	several	0.4	
Epidote		+	(CaCa)(AlAlFe ^3+^)O[Si _2_ o_7_][SiO _4_](OH)	0.4	
Sillimanite, Andalusite or Kyanite		+	Al _2_(SiO _4_)O	0.9	
Zircon		+	Zr(SiO _4_)	0.3	
Rutile		+	TiO _2_	0.3	
Jarosite		+	Kfe _3_(SO _4_) _2_(OH) _6_	0.3	
Apatite (Hydroxyapatite)		+	Ca _5_(PO _4_) _3_OH	0.4	
Titanite		+	CaTi(SiO _4_)O	0.4	
Granate		+	sevaral	0.3	
Galena		+	PbS	0.3	
Smectite		+	Na _0.409_K _0.024_Ca _0.009_(Si _3.738_Al _0.262_)(Al _1.598_Mg _0.214_Fe _0.173_Fe _0.035_)O _10_(OH) _2_	0.5	
Ca-Vermiculite		+	Ca _0.43_Mg _3_Si _3.14_Al _0.86_^ bn^_10_(OH) _2_	0.4	

For the aqueous-phase geochemical calculations, only the main mineralogical phases were taken into consideration, while in the Table 1. The impurities of the area are given for the sake of completeness. With this approach it is believed that the main geochemical interactions that could occur in the Marismas DHF are represented, however, as the heterogeneity of rock microstructure is possible even in microscale environments, the existence of additional geochemical reactions cannot be excluded. As calcite and aragonite are polymorphs, their presence was represented in the calculations by their common empirical formula given in the software as calcite mineral.

### Reservoir features

**Figure 2.**
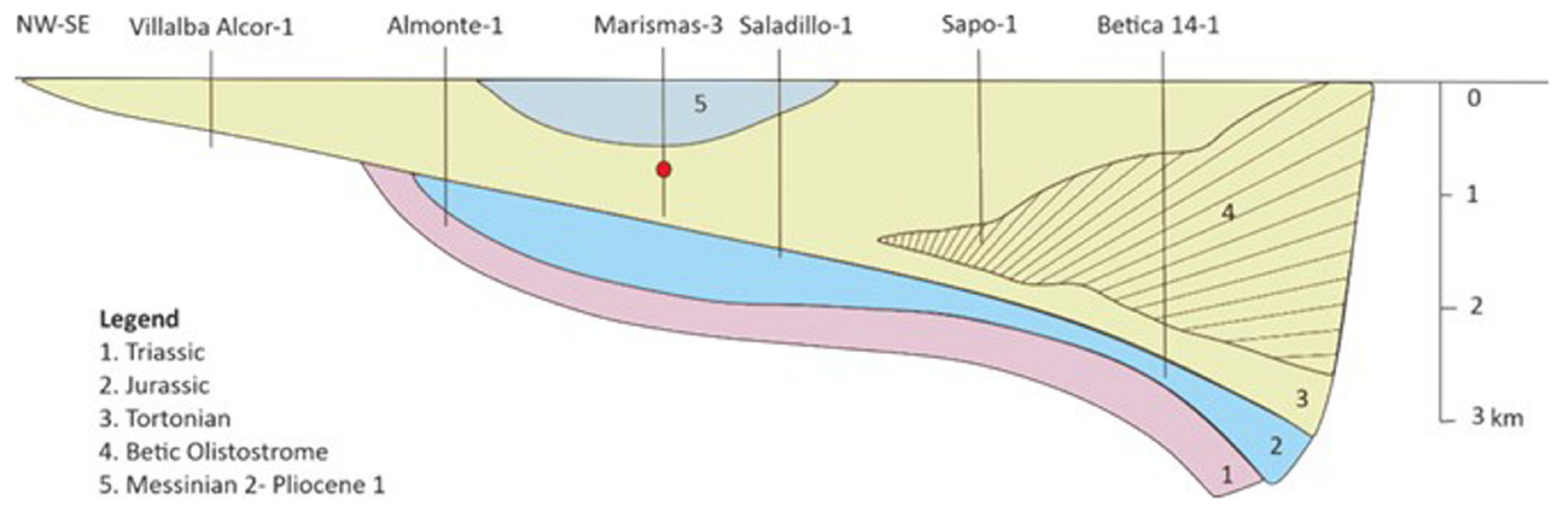
Simplified schematic cross-section over the Guadalquivir Basin and the Gulf of Cádiz (based on 29, 32).

Chevron discovered the Marismas fields in 1982, which produced gas since 1990. The average depth of the reservoirs ranges from 850 to 1000 m ^28^. In this study, Marismas 3 field ( Figure 2) was selected for further investigation with a depth of almost 1000 m ^29^ and an estimated volume of 7 × 10 ^8^ m ^3^ ^30^. Since this field is still under exploration, some data remain confidential. However, for the sake of the aqueous geochemical calculations presented in this report, some assumptions were made about the gas behavior.

The pressure response can change during the gas production from a field, separating the fields in three broad categories depending on the existence or absence of aquifer support as it is described below ^31^:

1. *Type 1 – No aquifer support*: pressure depletes almost linearly with the increasing produced gas. The recovery can exceed 90 % while a very low abandonment pressure occurs.

2. *Type 2 – Weak or limited aquifer support*: pressure is reduced regarding the gas production and that is reduced by small amount of water influx. The recovery is almost 70 % with critically higher abandonment pressures.

3. *Type 3 – Strong aquifer support*: the pressure decrease during the production phase is quickly replenished from the aquifer. The recovery is around 40 %.

Type 1 and 3 are favourable for the CO _2_ storage, while in Type 2 problems may occur as some initial capacity will be present when compressing up the remaining hydrocarbon gas. Subsequently, the additional capacity will change at the rate at which the aquifer relaxes in terms of CO _2_ injection, which may be too low for practical application ^31^.

In the Marismas area, the main Almonte-Marismas aquifer (Doñana aquifer) is at shallow depths ranging from 140 to 170 m; thus, strong aquifer support is lacking. As the depleted-gas reservoirs have, on average more than 15 % residual gas saturation as it was referred to in the literature, it was assumed that Marismas 3 belongs to Type 1 type with recovery close to 90 %. The abandonment pressure of such type depleted hydrocarbon reservoirs is assumed to be 2 MPa ^31^.

For the investigation of boundary conditions, a depth of 1000 m was considered. For simplicity, the temperature inside the reservoir was calculated assuming a linear geothermal gradient and homogeneous thermal properties of the sedimentary cover based on the following equation:

$$T=T_{surface}+\frac{\Delta T}{\Delta h}\times h\;[1]$$

where $\frac{\Delta T}{\Delta h}$ is the geothermal gradient and *h* is the reservoir’s depth. The mean land surface temperature over the year is almost 18 °C for the Marismas fields area ^33^. As the geothermal gradient is known and equal to 30 °C/km, the expected average temperature according to Equation 1 was calculated as 48 °C.

The porosity of the field is 20 % on average and the residual water saturation is 25 % ^34^. The residual hydrocarbon gas is composed by 98 % of CH _4_ and thus it was assumed to be the dominant hydrocarbon (100 %) for the run of the calculations. The initial water density was 1.04 and its pH was 7.02, while its temperature is assumed to be in equilibrium with the reservoir temperature. Based on these values, the activity of electrons (p _e_) in the investigated aqueous solutions was determined based on the following calculation:

$$p_{e}=\frac{E_{h}}{0.0001983\times T}\;[2]$$

where *E _h_* is the redox potential and T the temperature (K). Thus, for a temperature of 48 °C = 321.15 K, the equation becomes:

$$\;p_{e}=\frac{E_{h}}{0.0637}\;[3]$$

**Figure 3.**
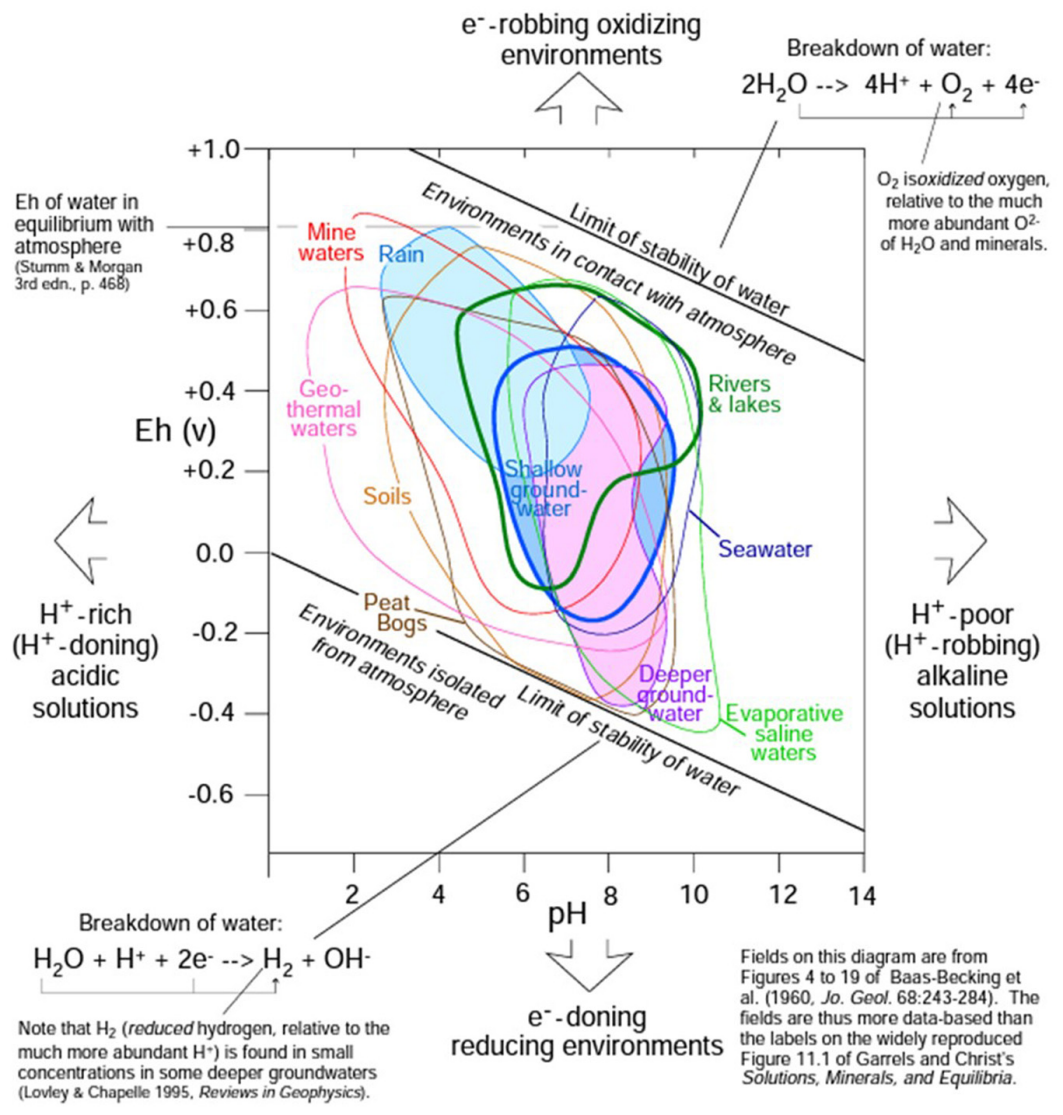
Characterisation of solutions by pH and Eh (figure has been reproduced with permission from Railsback ^37^).

Based on the graph in Figure 3, the *E _h_* is close to -0.18 V for an environment of deeper ground-water with the specific features given above. Therefore, *p _e_* is equal to -2.68, a reasonable value between the typical range of -12 to 25 *p _e_* water values. Moreover, to represent the real water characteristics in Marismas field, the calculated salinity of the field was assumed to be the alkalinity of the solution, as given in the Table 2.

**Table 2.** Residual water characteristics of the studied depleted gas field.

Properties	Water characteristics
Temperature (°C)	48
Pressure (MPa)	2
pH	7.0
p _e_	-2.68
Density (kg/m ^3^)	1040
Water mass (kg)	364 × 10 ^8^
n (mol)	2020507036249 *
Alkalinity (ppm)	35000
Volume (m ^3^)	35 × 10 ^6^

* Throughout the report the number of moles and other chemical quantities are provided in this format so that accuracy is not lost if attempting reproducibility of the results.

The concentration of the initial components in the reservoir was specified based on the Marismas field porosity (20 %), residual water saturation (25 %), and the reservoir volume which is 7 x 10 ^8^ m ^3^ ^30^. Thus, the residual water was calculated as 35 x 10 ^6^ m ^3^ and the free volume of the reservoir was up to 105 × 10 ^6 ^m ^3^. In Table 2, the water properties of the Marismas field used for the PHREEQC calculations are summarised.

Based on the available literature, the depleted-gas reservoirs have more than 15 % residual gas saturation on average ^5^. For the calculation of the residual gas in the reservoir that percentage was adopted and as CH _4_ was the dominant residual gas (up to 98 %), 21 × 10 ^6^ m ^3^ CH _4_ assumed to occupy the reservoir. The presence of other residual components was assumed as negligible. The CH _4_ temperature should be equal to the reservoir temperature while the brine-methane mixture should be the abandonment pressure for depleted hydrocarbon gas reservoirs, equal to 2 MPa, as referred in the literature ^35^. The CH _4_ density was found based on the Natural Gas Density Calculator and all the necessary CH _4_ properties for the run of the PHREEQC models are given in the Table 3.

**Table 3.** Residual gas (CH _4_) characteristics of the studied depleted gas field.

Properties	CH _4_ characteristics
Temperature (°C)	48
Density (kg/m ^3^)	12.33
Pressure (MPa)	2
Volume (m ^3^)	21 × 10 ^6^
m (kg)	25.893 × 10 ^7^
n (mol)	16142768080 *

* Throughout the report the number of moles and other chemical quantities are provided in this format so that accuracy is not lost if attempting reproducibility of the results.

During the CO _2_ injection in the DHF implemented for the CEEGS technology, the purity of CO _2_ was assumed to be 100 %. Based on CEEGS design, the injection of CO _2_ was planned in supercritical conditions, with temperature above 31°C, pressure above 7.38 MPa, and critical density 411.53 kg/m ^3^ ^36^. For the calculation of the CO _2_ injected moles in the reservoir, that critical density was used and the amount of moles kept constant in the reservoir despite the conditions’ changes. A deterministic modelling procedure was used to estimate CO _2_ storage capacity, taking into consideration the geological parameters of the selected area. Thus, the following equation referring to the Depleted Gas Hydrocarbon Fields was used ^4, 38^:


$${\text{M}}_{\text{C}{\text{O}}_{2}}=[\text{OGIP}\times \text{Bg}]\times \text{DF}\times \rho_{\text{RES}}\times {\text{E}}_{\text{GAS}}\;[4]$$

where

$$\text{OGIP}\times \text{Bg}=\text{A}\times \text{H}\times {\text{Φ}}_{\varepsilon }\times 1-{\text{S}}_{\text{W}1}\;[5]$$

and Equation 5 becomes

$${\text{M}}_{\text{C}{\text{O}}_{2}}=[\text{A}\times \text{H}\times {\text{Φ}}_{\varepsilon }\times 1-{\text{S}}_{\text{W}1}]\times \text{DF}\times {\text{ρ}}_{\text{RES}}\times {\text{E}}_{\text{GAS}}\;[6]$$

where M _CO _2__ is the mass of the CO _2_ storage capacity of a prospect field (kg), OGIP is the original gas in place at standard conditions, Bg is the formation volume factor of gas (%), DF is the depletion fraction as a percentage of OGIP, ρ _RES_ is the density of CO _2_ at reservoir storage conditions (kg/m ^3^), E _GAS_ is the storage efficiency factor (%), A is the total area of the reservoir (m ^2^), *H* is the gross reservoir thickness (m), Φ _ε_ is the effective porosity of the reservoir (%), and 1 – S _W1_ is the hydrocarbon saturation, i.e. 1 – water saturation (%).

In Marismas 3 case, the volume of the field is A × H = 7 × 10 ^8^ m ^3^ [7], Φ _ε_ is 20 % and S _W1_ is 25 %. DF is 85 %, and the remaining hydrocarbon gas based on the literature is assumed to be 15 %. ρ _RES_ is equal to the CO _2_ density in the supercritical state, and the E _GAS_ is typically in the range of 1.0 tο 5.0 % ^39^. As the storage efficiency factor is unknown for the Marismas field, both extreme values were used for the estimation of the G _CO _2__ creating to extreme scenarios. Finally:

$$367290525\;\text{kg}\;\text{<}\;{\text{M}}_{\text{C}{\text{O}}_{2}}<1836452625\;\text{kg}\;[8]$$,

and

$$\text{V}=\frac{\text{m}}{{\text{ρ}}_{\text{RES}}}\Leftrightarrow 892500\;{\text{m}}^{3}\;\text{<}\;\text{V}<4462500\;{\text{m}}^{3}\;[9]$$

The above assumptions were used as representative and fed the PHREEQC software ^1^ for subsequent calculations. The CO _2_ parameters are summarised in Table 4.

**Table 4.** Characteristics of the potential injected CO _2_ in the studied depleted gas field.

Properties	CO _2_ characteristics
Temperature (°C)	31.00
Pressure (MPa)	7.38
Density (kg/m ^3^)	411.53
Volume (Marismas 3) (m ^3^)	892500 – 4462500
m (kg) *	367290525 – 1836452625
n (mol) *	8345615201 – 41728076006

*Throughout the report the number of moles and other chemical quantities are provided in this format so that accuracy is not lost if attempting reproducibility of the results.

### PHREEQC numerical modelling tool

PHREEQC ^1^ is a computer simulation software written in C programming language that aims to perform a wide range of aqueous geochemical calculations based on PHREEQE Fortran program ^40^ completely rewritten to give new capabilities. PHREEQC design is based on an ion-association aqueous model and has potential for:

 i. speciation and saturation-index calculation, ii. Calculations for advective-transport and reaction-path, involving specified irreversible reactions, mixing of solutions, mineral and gas equilibria, ion-exchange reactions, and surface-complexation reactions, and iii. inverse modelling counting the transfers of a set of mineral and gas moles to investigate composition differences between aqueous solutions under specified compositional uncertainties ^41^.

PHREEQC software output values include the elements’ concentrations, aqueous species molalities and activities, phase mole transfers to achieve the equilibrium, pH, p _e_, and saturation indices (see extended PHREEQC output raw data) ^41^. For the relation of mass actions equations to actual solutions, known also as balanced chemical reactions, chemical thermodynamics, or specifically equilibrium thermodynamics are used. The basic principle to be achieved is that elements, molecules or compounds contain some internal energy and the whole system try to reach a state of minimum energy (equilibrium). Natural systems cannot always reach this state; however, they have this tendency ^42^.

PHREEQC has been extensively applied for the investigation of underground gasses’ storage systems aiming to explain the geochemical interactions between reservoirs rocks, the injected gasses to be stored (e.g. H _2_ or CO _2_), and the aquatic phases present in the reservoir, e.g., brine and/or residual hydrocarbons ^43, 44^.

In the present study, the following assumptions were taken for the calculations:

1. Only the chemistry of the main minerals comprising the host rock (reservoir) was considered.

2. Water and methane found in the reservoir pore volume supposed to be in chemical equilibrium with the reservoir’s minerals.

3. The CO _2_ injection occurs in the supercritical state, reaching pressure equilibrium with the rock-water-methane system.

4. The injected CO _2_ is assumed to transmit to gas phase during i. its route to the reservoir and ii. in the reservoir when micing with methane, acting as an ideal gas.

The accuracy of the aqueous geochemical interactions was verified by the estimation of new mineralogical phases available in the *phreeqc.dat* that were in agreement with the known impurities of the area given in Table 1.

The dataset *phreeqc.dat* contains the default thermodynamic data of PHREEQC as was derived from PHREEQE ^40^ with limitation regarding the smaller set of elements, aqueous species, and minerals ^45^. The latest *phreeqc.dat* characteristics included;

1. the parameters needed for the Peng-Robinson equation of state to calculate gases’ fugacity coefficients in a gas mixture and their solubility in water,

2. Solids’ and aqueous species’ molar volumes to calculate the pressure dependence of log K _s_ (applicable up to ~100 MPa and 200 °C, where *K _s_* is the solubility constant), and

3. the Redlich-type equation for temperature and pressure dependence of aqueous species’ volume ^46, 47^.

However, there is a limitation regarding the set of aqueous species, minerals, and gases that include the available parameters for corrections to ~200 °C. Some reactions do not include the parameters for calculating log K _s_ above 25 °C, while for others, only the enthalpy of reaction is available, and the estimation of logK _s_ is performed by the use of van ‘t Hoff equation ^48^ extrapolating from 25 °C to higher temperatures and assuming that enthalpy of reaction is invariant. This may result in relatively large uncertainties when higher temperatures are used ^49– 51^. In this study, only low temperatures were used and thus, the large uncertainties were avoided.

### PHREEQC scenarios and input values

Four different scenarios were developed for the PHREEQC calculations, separated in two sets. The two scenarios of each set are based on the boundary conditions of the potential injected moles of CO _2_, as given in Table 4, reflecting the 1.0 tο 5.0 % range of the storage efficiency factor (SEF _GAS_) ^39^. In the first set of scenarios, it is supposed that the CO _2_ is injected in the supercritical state and transmit to gas phase during its route to the reservoir and before interacting with the remaining hydrocarbons, while in the second set of scenarios it is supposed that the CO _2_ is in gas phase when gets into equilibrium with the remaining hydrocarbons in the reservoir. All the calculations were time-independently as the purpose was the systems to reach the chemical equilibrium between their initial components. For all developed scenarios, the *phreeqc.dat* database was used with addition of glauconite mineralalogical phase from *sit.dat* to allow for comprehensive main mineralogical phases’ consideration. The whole calculations were run with *phreeqc.dat*. Αs *phreeq.dat* take into consideration all the potential aqueous chemical reactions between the mineralogical phases of the database in the background of the software and thus they are not provided to the user. Table 5 provides details for the selected databases, fitting to the requirements of the present study. The *sit.dat* uses specific ion interaction theory (SIT) for calculations in concentrated solutions and is suitable for a range of conditions expected in both geological disposal facilities and near-surface conditions (i.e. pH 6–14, ionic strength up to SIT limit, redox potential (E _h_) within the water stability fields, and 15 to 80 °C temperatures ^52^.

**Table 5.** Thermodynamic data characteristics for each database used in this study, based on 45.

Database	T-P range	Corrected P range	Aqueous activity coefficient model	Fugacity coefficients	Species’ number	Limitations	Reference
phreeqc.dat	<200 °C, <100 MPa	up to ~ 100 MPa	mixed WATEQ and Davies equation	Peng-Robinson	~310	*•* Relatively small species’ number *•* Ionic strength generally less than one molal	
sit.dat	15–80 °C at 100 MPa	N.A.	Specific Ion Interaction Theory	Ideal gas law	~2300	*•* Small T-P range *•* C = logKs extrapolation using the van ‘t Hoff equation *•* Ideal gas law for gas fugacity coefficients *•* No pressure correction *•* Ionic strength generally less than one molal	Amphos 21, French Bureau of Research for Geology and Mining and HydrAsa for French Agency for the Management of Nuclear Waste based on ThermoChimie Version 9b0 ^45^

All scenarios included a common first step of calculations, including the host rock/methane/water interactions, followed by the second step, which reflected the CO _2_ injection and thus the host rock/methane/water/CO _2_ interactions. The input values for each scenario are extensively described below.

***Set 1-Scenario 1***

As the main lithologies of the Marismas fields area are carbonate-silisiclastic rocks and sands, they have densities of 2.15-2.40 × 10 ^3^ kg/m ^3^ and 1.60-2.00 × 10 ^3^ kg/m ^3^, respectively ^53^. Thus, an average density is almost 2.04 × 10 ^3 ^kg/m ^3^. The reservoir volume is known by the literature and is estimated to be 7 x 10 ^8^ m ^3^ and the porosity of the reservoir is 20 %, a total volume of 80 % has a mass of 11424 × 10 ^8^ kg ( Table 6).

**Table 6.** Masses of components interacted in the studied depleted gas field.

Components	Mass (kg × 10 ^8^)
Rock	11424
Water	364
CH _4_	2.5893
CO _2_	3.67290525 - 18.36452625

The mass of the main mineralogical phases was calculated based on the percentages’ estimation given in Table 1, presenting as raw data in the Table 7. The raw data were used for the calculation of moles of initial components provided as input values for PHREEQC calculations ensuring quality importance. The CH _4_ of the system was assumed to behave as an ideal gas for the purpose of simulations.

**Table 7.** Quantities of mineralogical phases in the host rock of depleted gas field. Only the main mineralogical phases were used after normalisation of the amounts in 100 %.

Mineral	Initial Estimation (in 100 %)	Mass in rock (× 10 ^6^ kg)	Empirical formula based on the used databases	Molecular mass (g/mol)	Moles × 10 ^3^
Calcite or/and Aragonite	29	331296	CaCO _3_	100	3312960000
Dolomite	10	114240	CaMg(CO _3_) _2_	184	620869565
Quartz	29	331296	SiO _2_	60	5521600000
Alkali Feldspars	2	22848	KAlSi _3_O _8_	278	82187050
Muscovite	2	22848	KAl _3_Si _3_O _10_(OH) _2_	398	57407035
Illite	3	34272	K _0.6_Mg _0.25_Al _2.3_Si _3.5_O _10_(OH) _2_	384	89250000
Kaolinite	9	102816	Al _2_Si _2_O _5_(OH) _4_	258	398511628
Chlorite	2	22848	Mg _5_Al _2_Si _3_O _10_(OH) _8_	556	41093525
Glauconite	14	159936	K _0.75_Mg _0.25_Fe _1.5_Al _0.50_Si _3.75_O _10_(OH) _2_	432	370222222
Total	100	1142400			

The rock mass and the stoichiometry of the main mineralogical phases given in Table 7 were used to specify the number of moles interacted in the system for the PHREEQC calculations.

To save computational time and avoid computational limitations posed by PHREEQC software, it was assumed that the whole system is composed of 10000 moles. The ratios between the system’s initial components are kept stable to represent reality. In this scenario, the minimum ratio of storage efficiency factor, which means 1.0 % was used ( Table 8). For the gas phases (i.e., CH _4_ and CO _2_) used in the calculations, the log of gas partial pressure was used as a target saturation index. This may not be attained if the amount of the phase in the assemblage is insufficient. The Ideal Gas Law was used to calculate the partial pressure in the Partial Pressure Calculator ^54^ as the gasses were assumed to behave as ideal. In this case, the CO _2_ supposed to transmit into gas phase during the route to the reservoir keeping its initial temperature (31°C). Moreover, it was supposed that the two gasses are not yet mixed and thus the partial pressure of each one as a separate gas was calculated.

**Table 8.** Quantities of initial components in the complex system after the injection of CO _2_ as estimated and used for PHREEQC calculations for scenario 1.

Component	Moles × 10 ^3^	Moles in 10000 (used in PHREEQC)
Calcite or/and Aragonite	3312960000	2642
Dolomite	620869565	495
Quartz	5521600000	4404
Alkali Feldspars	82187050	66
Muscovite (K-mica)	57407035	46
Illite	89250000	71
Kaolinite	398511628	318
Chlorite	41093525	33
Glauconite	370222222	295
Water	2020507036	1611
CH _4_	16142768	13
CO _2_	8345615	7
Total	12514608061	10000

***Set 1 - Scenario 2***

This scenario is similar with Set 1 – Scenario 1 with the only difference in the used maximum storage efficiency factor ratio, which was equal to 5.0 %. To achieve comparable results, the other initial components are kept in the same ratios; thus, the whole system is composed of 10026 moles ( Table 9).

**Table 9.** Quantities of initial components in the complex system after the injection of CO _2_ as estimated and used for PHREEQC calculations for scenario 2.

Component	Moles × 10 ^3^	Moles in 10026(used in PHREEQC)
Calcite or/and Aragonite	3312960000	2642
Dolomite	620869565	495
Quartz	5521600000	4404
Alkali Feldspars	82187050	66
Muscovite (K-mica)	57407035	46
Illite	89250000	71
Kaolinite	398511628	318
Chlorite	41093525	33
Glauconite	370222222	295
Water	2020507036	1611
CH _4_	16142768	13
CO _2_	41728076006	33
Total	12572478906397	10026

***Set 2 – Scenario 1***

This scenario is the same as Set 1 – Scenario 2 with the only difference that the CO _2_ supposed to transmit into gas phase when interacting with the remaining hydrocarbons in the reservoir. Thus, the Ideal Gas Law was used to calculate the partial pressure of the gas mixture (CO _2_ and CH _4_) in the Partial Pressure Calculator ^55^. In this case, the CO _2_ and the CH _4_ supposed to have a common temperature of 48 °C as the temperature of the reservoir.

***Set 2 – Scenario 2***

This scenario is the same as Set 2 – Scenario 1 with the only difference in the used maximum storage efficiency factor ratio, which was equal to 5.0 %.

### CMG-GEM numerical modelling tool

CO _2_ injection and subsequent plume migration in the “Depleted Hydrocarbons Field (DHF)” scenario are modelled using the CMG-GEM compositional multi-phase flow subsurface simulator ^3^. The modelling of CO _2_ injection and back production involves the component transport equations solution, the solution of equations for thermodynamic equilibrium between the gas (CO _2_ and methane) and aqueous (formation water) phase, and the geochemical reactions solutions. The solution adopted in GEM for CO _2_ transport-reaction simulations for porous media reservoirs is the flow simulation for adaptive-implicit multiphase multicomponent under phase and chemical equilibrium and rate-dependent mineral dissolution/ precipitation by the use of the fully-coupled approach ^56, 57^.

### CMG-GEM model description and input values

**Figure 4.**
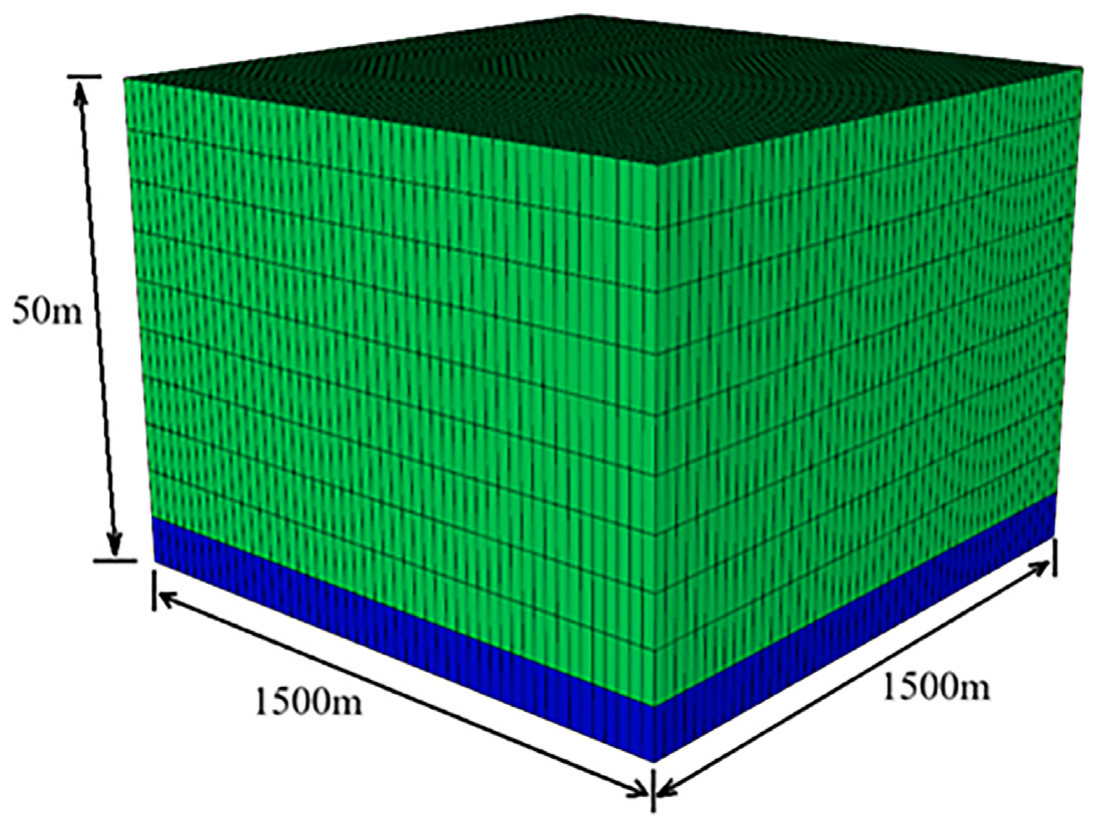
Water saturation in the depleted hydrocarbon reservoir (blue grids: water saturation 100%, green grids: water saturation is 25%).

A more generic, small-scale model of natural gas reservoir for a partially closed depleted gas reservoir with an underlying aquifer was developed. By introducing the element of an underlying aquifer in the system allowed to make a more complex approach possible to represent a wider range of areas of interest. According to Carneiro and Behnous guidelines ^58^, the minimum pressure required for a reservoir at a depth of 2000 m to ensure the production of supercritical CO _2_ in the CEEGS technology, should exceed 17 MPa. In CMG-GEM simulation, the targeted reservoir is located at a depth of 2000 m in contrast to Marismas field depth which was 1000 m, starting with an initial pressure of 14 MPa and a temperature of 75°C (with a normal geothermal gradient of 30 °C/km). The reservoir exhibits a water saturation of 25%, and the remaining gas composition comprises 98% CH _4_, 1% C _2_H _6_, 0.5% C _3_H _8_, and 0.5% CO _2_. Water was assumed to have flow back to the reservoir, following production of the gas, and is virtually the single existing phase in the deepest parts of reservoir. The reservoir dimensions are specified in this more generic scenario as 1500m x 1500m x 50m (see Figure 4). Detailed information on other reservoir parameters are given in Table 10.

**Table 10.** Parameters of the depleted reservoir simulation model.

Parameter	Value
Reservoir size	1500m x 1500m x 50m
Depth to reservoir top (m)	2000
Porosity (%)	20
Permeability (md)	200
Thickness (m)	50
Reservoir initial temperature (°C)	75
Reservoir initial pressure (MPa)	14
Residual water saturation (-)	0.25
Residual Gas saturation	0.3
Gas water contact (GWC) (m)	2040
Natural gas composition (mole fraction)	98% CH _4_, 1% C _2_H _6_, 0.5% C _3_H _8_, and 0.5% CO _2_
Injected gas rate	33 kg/s of pure CO _2_
Top and bottom boundary conditions of the reservoir	No fluid flow and no heat flux

The reservoir consists of two wells, designated as A and B. To inject CO _2_ into the depleted natural gas reservoir while minimizing water upconing at the production well, the process begins with a CO _2_ plume setup phase. During this phase, CO _2_ is continuously injected at a rate of 33 kg/s (equivalent to 1 Mtonne/year) for two years to establish a supercritical CO _2_ plume. Once the initial setup is complete, the second stage involves energy storage through charge-discharge cycles. In the charge phase, surplus renewable electricity is used to compress, heat, and inject CO _2_ into the reservoir via Well A. During the discharge phase, Well A produces supercritical CO _2_, which is utilized in the surface components of the CEEGS system to generate electricity. Meanwhile, Well B reinjects CO _2_ into the reservoir at the same flow rate as Well A but at a lower temperature. In brief, this scenario involves two years of continuous injection to establish the CO _2_ plume, followed by a one-month shut-in period. Additionally, six cycles of charge-discharges are included in the analysis. 

The initial CO _2_ plume setup plays a critical role not only in the economic feasibility of the system but also in determining the relative contributions of CO _2_ permanent sequestration, energy storage, and geothermal heat extraction.

During the charge phase, Well A injects CO _2_ at a wellhead temperature of 60°C, while Well B remains inactive. In the discharge phase, Well A produces CO _2_, which is subsequently reinjected through Well B at the same flow rate but at a reduced temperature of 20°C. The injection process is subject to two key constraints: the bottomhole pressure must not exceed 20% of the initial hydrostatic pressure, and sufficient injection pressure is required to deliver CO _2_ to the bottom of the reservoir. The cycle of charge, shut-in, and discharge was repeated six times.

## Results and discussion

### PHREEQC calculations

***Initial conditions***

All the developed scenarios of the present study have a common first step including investigating interactions between the reservoir rock/residual water/CH _4_ of the Marismas 3 field. The pH of the aqueous solution in the reservoir before the injection changes from the initial water pH of 7.0 to 9.6, becoming more alkaline. This alkaline nature can be explained by the ions’ presence in the reservoir, i.e. Al, K, Ca, and Mg. The pH change is the result of a charge balance. The p _e_ parameter was also changed to -7.26 adjusting to redox equilibrium. Among the main mineralogical phases of the reservoir host rock, i.e. calcite, dolomite, quartz, K-feldspars, K-mica, illite, kaolinite, chlorite, and glauconite, only the K-feldspar and illite were unstable. This can be explained by the gradual complete dissolution of K-feldspar, resulting in the precipitation of a variety of new mineralogical phases after long period of time ^59^. In such conditions, gibbsite (Al(OH) _3_), K-mica, and kaolinite are some of the mineralogical phases that can occur after the dissolution of K-feldspar ^60^. However, the precipitation rate of kaolinite is slower than the dissolution of the feldspar, leading to kaolinite-supersaturated solutions in the reservoir ^61^. Illite dissolution is more selective and occurs only under alkaline pH conditions, at temperatures 100 °C and above, while when the pH decreases below 5, a rapid precipitation of the phase of aluminum oxy(hydroxide) is expected ^55^. However, in this case study, the system’s newly formed minerals containing aluminum were undersaturated. This means that despite the lack of *phreeqc.dat* database in this phase and the computational limitations, there is no possibility of such phase precipitation. A change in the K-mica interacting moles (from 46 to 155) was observed due to the dissolution of K-feldspar and the precipitation of newly formed K-mica.

The other main mineralogical phases of the reservoir were in equilibrium with the system. The reservoir was supersaturated in hematite (Saturation Index-S.I. 11.48), goethite (S.I. 4.68), siderite (FeCO _3_) (S.I. 3.50), and talc (Mg _3_Si _4_O _10_(OH) _2_) (S.I. 1.48). The presence of hematite and goethite agreed with the impurities identified in the study area as given in Table 1, owing their origin in glauconite transformation ^62^. Glauconite can also alter K-mica ^63^ and precipitate siderite by diagenetic conversion ^64^. A main difference among glauconite and siderite is the presence of mostly oxidised Fe ^3+^ in the first one, and the presence of reduced Fe ^2+^ to the second one ^63^. Talc (Mg _3_Si _4_O _10_(OH) _2_) precipitation can be attributed to i. sedimentary magnesium carbonate rocks alteration at elevated pressure and temperature, and/or ii. magnesium-rich ultramafic rocks alteration under high pressures and temperatures up to 700 °C. The second origin is not possible in this study area, as the reservoir temperature is critically lower. Thus, talc can origin by chlorite + quartz = kyanite + talc + water ^65^.

***Set 1 - Scenario 1***

The second step of PHREEQC calculations includes the investigation of interactions between rock/water/CH _4_ system after the injection of CO _2_. The pH critically decreases after the CO _2_ injection, changing from 9.6 to 7.6 due to the dissolution of CO _2_ in the water, which starts as soon as the CO _2_ is injected ^66^. Due to buoyancy, the CO _2_ flows to the top of the reservoir. In this upward flow, an amount of CO _2_ partly reacts with the water becoming carbonic acid (H _2_CO _3_). This amount is generally small due to the small available storage capacity ^67^ and the water availability in the system. The Total CO _2_ increased from a value of 0.46 mol/kg for the rock/water/CH _4_ system to 0.69 mol/kg due to the CO _2_ injection, as it was expected. This parameter represents the Dissolved Inorganic Carbon (DIC), without being restricted to CO _2_ but also including HCO _3_, ${\text{CO}}_{3}^{2-}$ , and other carbon-based complexes as these provided by each run of software’s simulation. The p _e_ parameter was equal to -4.94, adjusting to redox equilibrium. The CO _2_ gas completely dissolved in the reservoir solution when reached the equilibrium, significantly decreasing its pressure from 7.38 MPa to 0.11 MPa, which is related to the change of the temperature and its equilibrium with the brine-methane system having an initial pressure of 2.00 MPa. As the system comes to equilibrium, the system pressure stabilises and the CH _4_ pressure will slightly decrease to 1.93 MPa.

In the case of rock/water/CH _4_ a complete dissolution of the illite was observed. In contrast, when CO _2_ is injected in the system, the illite not only is not dissolved but it is precipitated (S.I. 0.36). This behavior can be explained by the complete K-feldspar dissolution resulting in quartz and clay precipitation under abundant CO _2_ presence at underground P-T conditions, as it was verified by experimental data available in the literature ^68^. Moreover, the CO _2_ injection in a reservoir containing K-feldspar increases significantly its dissolution rate as the initial equilibrium of the reservoir with the containing fluids is disrupted. However, the remaining fluids are then supersaturated to kaolinite approaching the previously established equilibrium, resulting in relatively low dissolution rates for the Al-Si minerals, as it was proved by experimental data Thus, the rhythm of secondary products precipitation is controlling the K-feldspar dissolution rate as it allows the fluid to approach saturation with K-feldspar than secondary products ^61^. As the secondary minerals can control the dissolution of K-feldspar, its complete dissolution and the reach of the reservoir in chemical equilibrium can be a process that take a long period of time. The supersaturation of quartz (S.I. 0.49), chalcedony (S.I. 0.13), kaolinite (S.I. 5.04), K-mica (S.I. 5.58), and gibbsite (S.I. 1.57) can be also explained by this process. Another important differentiation in the system after the CO _2_ injection, which is in agreement with the literature ^68^, is the carbonate minerals dissolution, with S.I. -0.68/-0.87 and -1.60 for calcite/aragonite and dolomite minerals, respectively. A series of reactions take place after the CO _2_ injection into the solution, starting from the CO _2_ dissolution in the water which forms H _2_CO _3_ acid. This acid is diluted in the aqueous solution of the reservoir causing a pH drop and an acid attack to the rock minerals. The following reactions with the carbonate minerals explain the less acidic nature of the brine:

$$\text{C}{\text{O}}_{2}+{\text{H}}_{2}\text{O}\to {\text{H}}^{+}+\text{HC}{\text{O}}_{3}^{-}\;[10]$$

$$\text{CaC}{\text{o}}_{3}(\text{calcite})+{\text{H}}^{+}\to \text{C}{\text{a}}^{2+}+\text{HC}{\text{O}}_{3}^{-}\;[11]$$

$$\text{CaMg}{\left(\text{C}{\text{O}}_{3}\right)}_{2}+{\text{H}}^{+}\to \text{C}{\text{a}}^{2+}+\text{M}{\text{g}}^{2+}+\text{HC}{\text{O}}_{3}^{-}\;[12]$$

The less acidic brine created by the CO _2_ injection, has the ability to dissolve minerals that contain divalent cations such as Mg, Ca, and Fe ^69^. Common minerals containing divalent cations are chlorite, montmorillonite, talc and glauconite ^70^. In this case, the dissolution of glauconite (S.I. -3.60), talc (-6.73), and chlorite (-11.42) were observed, while despite the partly dissolution of montmorillonite, other processes enhanced its precipitation. Thus, due to the dissolution of several mineralogical phases that extensively described above, as the rapidly dissolved calcite (S.I. -0.68) and to lesser extent dolomite (-1.60), and the prolonged dissolution of K-feldspar (-3.32) and chlorite (-11.42), liberated ions are present in the brine having the ability to react with each other resulting in clay minerals formulations, such as kaolinite (5.04), Ca-Montmorillonite (S.I. 3.85), and illite (S.I. 0.36). Hematite and goethite remain supersaturated also after the CO _2_ injection, with significantly lower values, i.e. S.I. 6.09 and 1.99, respectively. This decrease is owing to Fe ^2+^ release from the glauconite and chlorite dissolve which is oxidised to Fe ^3+^, resulting in such minerals’ precipitation. Amorphous phases of Fe(OH) _3_, Al(OH) _3_, and SiO _2_ are undersaturated in the system. Siderite (FeCO _3_) as a carbonate mineral is partly dissolutes, however, the dissolution of glauconite released Fe and the redox reaction of calcite and/or dolomite with H _2_CO _3_ acid, gives to the system the potential to form an amount of siderite (S.I. 3.08) which finally precipitates. Moreover, the dissolution rate of carbonates decreases in the following order: calcite > dolomite > siderite > magnesite ^71^, which proves the slower siderite dissolution than calcite and dolomite.

Permeability reduction in porous reservoir media is widely investigated during the CO _2_ injection ^72, 73^. In Marismas field, a first increase of the permeability could be observed during the CO _2_ injection, owing to the carbonates dissolution as it was referred in the literature from studies held in similar environments ^74^. However, potential decrease in the pressure result in the precipitation of the dissolute carbonate minerals or other secondary minerals, such as illite, kaolinite or Ca-montmorillonite that tend to migrate to pore throats decreasing the permeability ^75^. Moreover, the existing clay minerals of the reservoir may cause permeability reduction due to the release of clay particles from pore walls of the reservoir and their subsequent redeposition in the pore throats downstream, which potentially have smaller diameters than the pores ^76^. However, when chlorite is present in the reservoir, aluminum silicate minerals and chlorite itself are dissolved to some extent, minimising the precipitation of secondary carbonate minerals and improving the reservoir properties ^77^. In this study, a significant amount of clay minerals in precipitating, including illite, K-mica, kaolinite, and Ca-montmorillonite which could transfer in the fluid flow path and potentially accumulate at pore throats, reducing the permeability. Significant changes in the permeability can be due to the swelling clays ^75^. However, most clay minerals precipitated in the investigated system are non-swelling minerals, except Ca-montmorillonite. Ca-montmorillonite dissolution can be controlled by the control of temperature, pH, and time ^78^. In this case, where low temperatures exist in the reservoir, a change of pH in more alkalic or more acidic conditions favors the dissolution of the mineral to avoid clogging problems, as in almost neutral environment the mineral is in equilibrium ^78^. However, further investigation is needed.

The chemical precipitation of certain ions such as manganese (Mn) and iron (Fe) are extremely important in the process of chemical clogging. Simultaneously, it is strongly connected with the metal bacteria involvement that can enhance the chemical clogging. The partially oxidised and low crystalline intermediates of Fe (Fe ^2+^, Fe ^3+^) followed by stable crystalline Fe ^3+^(hydro)oxides (e.g. goethite) are the end products of iron redox reaction ^79^. The reduced precipitation of hematite minerals can ensure the goethite precipitation in a relatively stable form that is not correlated to the clogging of the pores ^80^. Special attention is required for the siderite’s precipitation. Its small colloidal particles remain suspended in the fluid, and the ability to transport over long distances and eventually accumulate in certain areas may lead to the reservoir's clogging or corrosion ^81^. The management of Fe-minerals precipitation can be easily controlled by changing the system's parameters and especially pH, pe, and oxygen concentrations to avoid failures in the reservoir ^79^.

Despite the constant attention that permeability requires during injection, the porosity of the investigated system finally increases after the CO _2_ injection, as the solution occupies a volume of 30.92 L instead of 30.66 L of rock/water/CH _4_ system.

***Set 1 - Scenario 2***

Scenario 2 gave similar results to Scenario 1 as simulates the same system with only difference the higher concentration of CO _2_ reflecting the maximum ratio of storage efficiency factor. Partial differentiations occurred in the system relative to higher CO _2_ amount. Firstly, the pH of the aqueous solution is getting more acidic after the higher injected CO _2_, changing from 9.6 to 6.0, a value lower than the Scenario 1 (pH 7.6) related to the CO _2_ partially reaction with the water creating carbonic acid (H _2_CO _3_). Thus, a higher percentage of carbonate minerals is expected to be dissolved. This phenomenon is more intense when the storage capacity of CO _2_ or brine quantity are increasing. When the CO _2_ reaches the top of the reservoir, the diffusion is the dominant process determining the dissolution rate. As this process is slow, the resulting dissolution rate is also low. However, when the dissolved CO _2 _increases, an increase in the brine density is also observed, creating an unstable buoyant layer below the CO _2_. When this phenomenon occurs and the layer becomes sufficiently unstable, a downward convective flow with a fingers’ shape is developed, causing an upwelling transport of fresh brine to the interface of CO _2_/brine that has the ability to increase the CO _2_ dissolution rate ^66^. Despite that in the Scenario 2 the storage capacity is significantly higher than in Scenario 1, as the CO _2_ was completely dissolved as a gas in the reservoir’s solution, it has not the ability to create an unstable buoyant layer that could significantly increase the carbonates dissolution causing potential stability problems of the reservoir. This complete CO _2_ dissolve in the reservoir’s solution as a gas caused a decrease in its pressure from 7.38 MPa to 4.53 MPa, critically higher than in Scenario 1 (0.11 MPa). The maintenance of pH values in values higher than 5, despite the increase of CO _2_ storage capacity, causing the dissolved carbon dioxide main transformation into bicarbonate ions, which will act as dissolution inhibitors later on, and based on the available literature ^82, 83^ may cause a greater reservoir stability than the one that was expected. The p _e_ parameter was increased to -3.14. The volume of the aqueous solution occupied in this Scenario was 31.84 L, slightly higher than in Scenario 1 (30.92 L) corresponding to the higher dissolution of carbonate minerals due to the CO _2_ increase, leading also to the increase of free space. As a result, the Total CO _2_ was also increased from a value of 0.46 mol/kg for the rock/water/CH _4_ system to 0.69 mol/kg for the Scenario 1 and to an even higher value of 1.56 mol/kg for the Scenario 2 where a higher CO _2_ concentration was injected. As the pressure of CH _4_ after the system reached equilibrium remained constant as in the Scenario 1.

Concerning the mineralogical phases of the system, slight changes occurred when the concentration of CO _2_ increased. An amorphous Al(OH) _3_ supersaturation occurred (S.I. 0.44) due to chlorite and K-feldspar dissolution. This wasn’t observed in the Scenario 1 due to complete crystallisation of Al(OH) _3_ to gibbsite (Al(OH) _3_) and clay minerals. As the Si-based mineralogical phases remained almost stable, there was a lack of free silicon to potentially create other aluminosilicate minerals containing an Al(OH) _3_ amount. In the present Scenario, common clay minerals were precipitated as in the Scenario 1. A differentiation was occurred in the precipitation of Fe-minerals, i.e. goethite (S.I. -0.87), siderite (S.I. 1.51), and hematite (S.I. 0.37), related to the pH and p _e_ changes that have the ability to dissolve the goethite that exist in the area as an impurity. This allows for the hematite and siderite precipitation to a lower extent. The less siderite percolates in the system, the better as it reduces the pore throat clogging effect.

***Set 2 – Scenario 1***

Scenario 1 of the second set gave almost the same results with Set 1 – Scenario 1, with only difference the CH _4_ pressure which was slightly lower than Set 1 -Scenario 1, equal to 1.80 MPa.

***Set 2 – Scenario 2***

Scenario 2 of the second set gave similar geochemical interactions with the Set 1 – Scenario 2. The general characteristics of the system kept constant. The saturation indices of the minerals were slightly differentiated, however, the minerals that were undersaturated kept undersaturated, e.g. anorthite (S.I. -2.65), aragonite (S.I. -2.28), calcite (S.I. -2.09), chlorite (S.I. -23.54), chrysotile (S.I. -19.84), dolomite (S.I. -4.46), glauconite (S.I. -8.81), goethite (S.I. -0.70), K-feldspar (S.I. -3.43), sepiolite (S.I. -14.02), and talc (S.I. -15.57), while the minerals that were precipitated had the same behavior, e.g. Ca-montmorillonite (S.I. 6.56), chalcedony (S.I. 0.15), gibbsite (S.I. 2.91), illite (S.I. 1.86), hematite (S.I. 0.72), K-mica (S.I. 8.15), kaolinite (S.I. 7.75), quartz (S.I. 0.51) and siderite (S.I. 1.59). In this case, the CO _2_ was not completely dissolved as a gas in the reservoir’s solution and its pressure was 3.59 MPa. The pressure of CH _4_ after the system reached equilibrium was 1.57 MPa.

### CMG-GEM calculations

**Figure 5.**
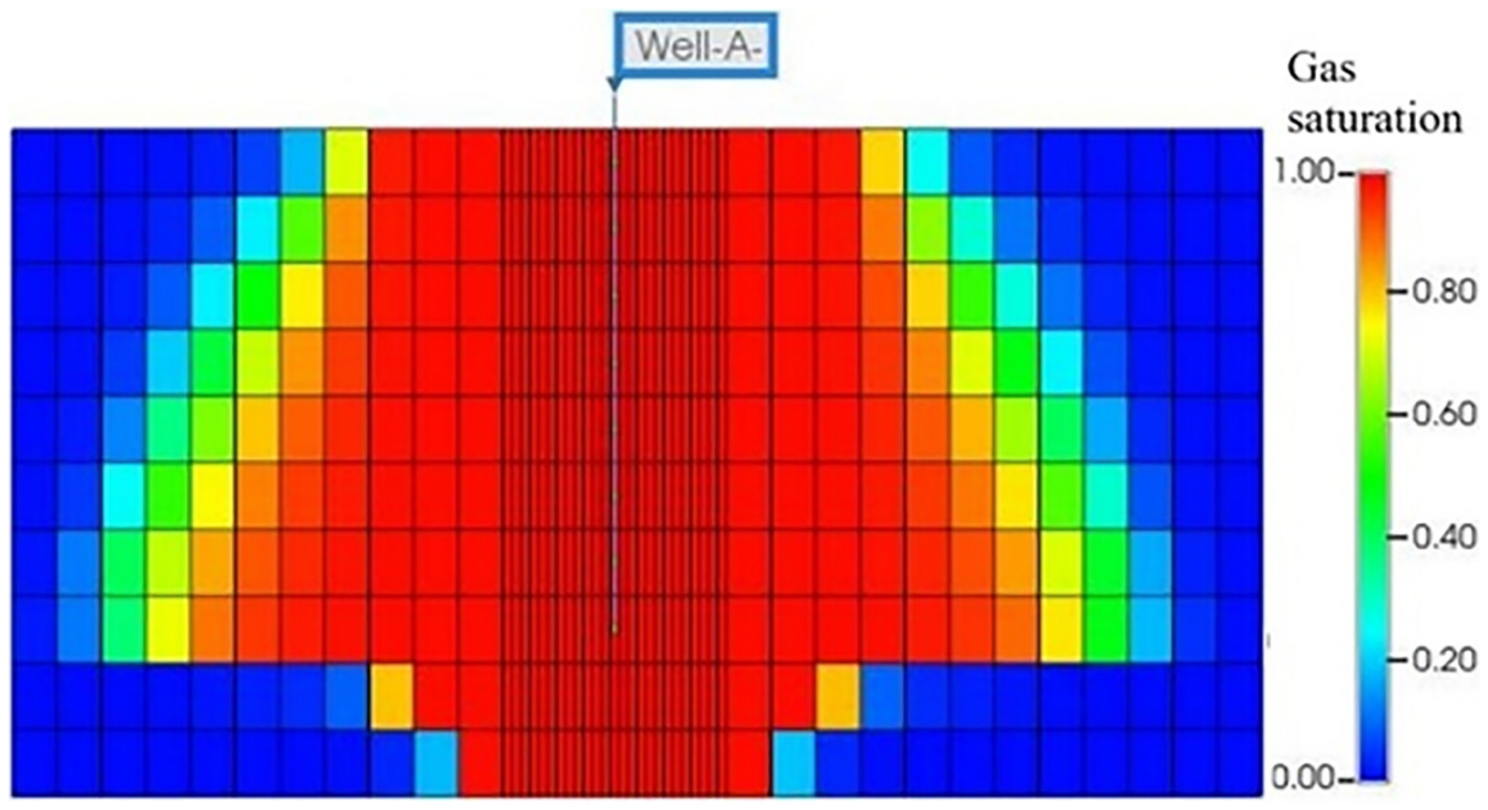
CO _2_ Plume distribution (Gas saturation) after 2 Years of Continuous Injection.

The development of the CO _2_ plume following two years of continuous injection is given in Figure 5. The plume takes on a distinct shape, taking to an upright cone. Notably, the maximum extent of the plume is noticeable in the lower section of the reservoir, near the point of separation between the aquifer and the gas. The plume's shape can be attributed to the higher density of CO _2_ in reservoir conditions (pressure and temperature) compared to impure methane (six times higher). A crucial finding is that for achieving optimal CO _2_ concentration, production is advised from beneath the horizontal centerline of the reservoir. In addition, as more CO _2_ is injected into the reservoir, a gas mixture of CO _2_ and CH _4_ is produced, where the CO _2_ fraction increases and the CH _4_ fraction decreases with time (Ezekiel *et al*., 2020). This may require additional fluid separation at the surface to obtain a high-purity supercritical CO _2_, making it suitable before entering surface components. However, this is disadvantageous as it could reduce the system’ efficiency.

**Figure 6.**
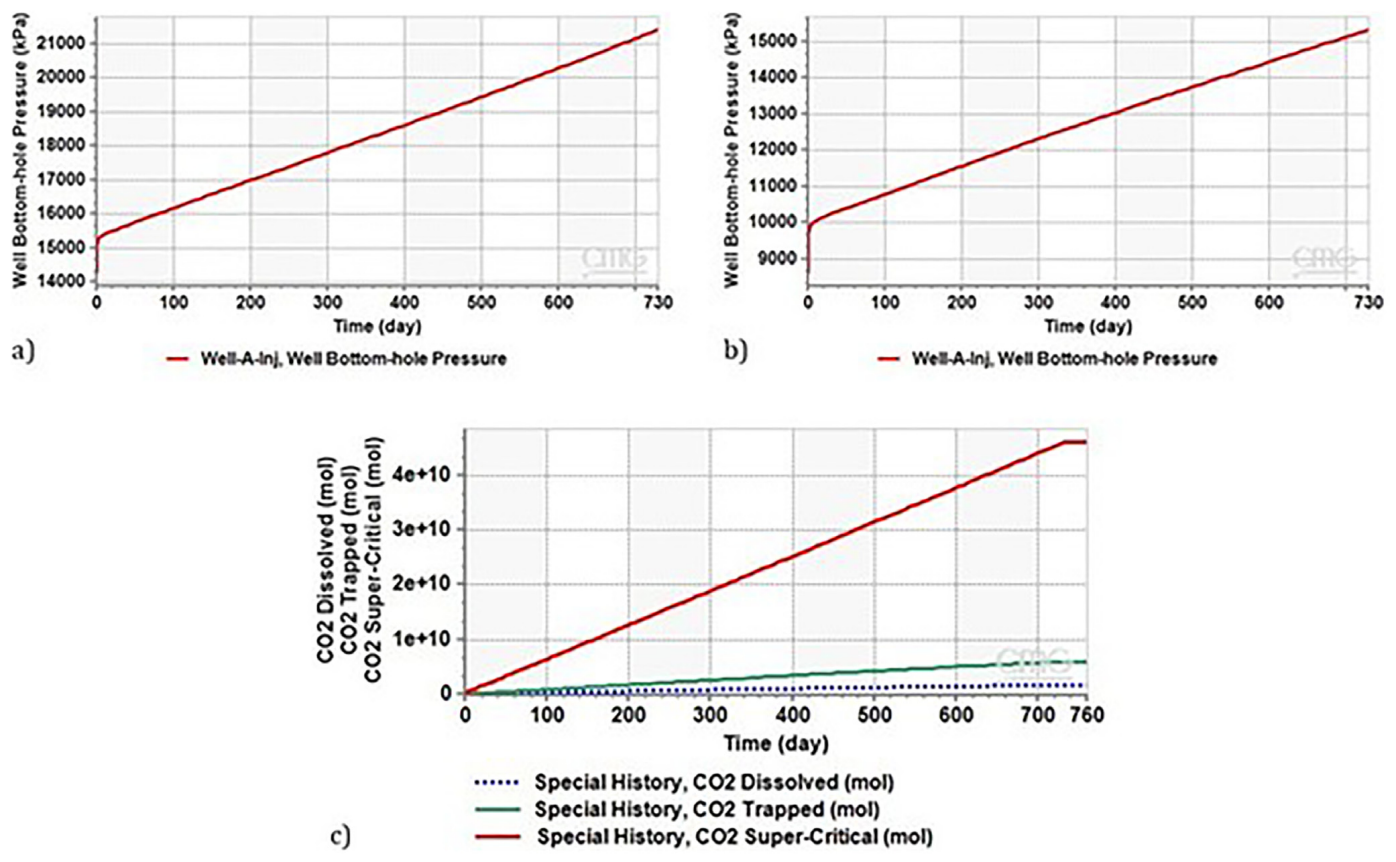
**a**) Evolution of Bottom Hole Pressure in Well A injector over a 2-Year Period, **b**) Evolution of Bottom Hole Pressure during Two-Year Continuous CO _2_ Injection in Depleted Reservoir (8MPa, 2000m), **c**) CO _2_ Distribution and Storage Dynamics during Plume Setup Phase for the case of a highly depleted reservoir, the simulated conditions referred to a reservoir pressure of 8 MPa at 2000 meters.

Figure 6a illustrates the evolution of bottom-hole pressure in the injection well (Well A) over a two-year period. Initially, before starting injection, the bottom-hole pressure is assumed to be 14 MPa. This pressure, commonly occurring in depleted reservoirs due to long-term gas production, does not allow for the production of supercritical CO _2_. However, after approximately two years of injection, equivalent to roughly more than two million tons of CO _2_, the pressure increased to 21.3 MPa. Importantly, this pressure remains below the threshold assumed to avoid fracturing the reservoir and seal, that is 20% increase above the hydrostatic pressure, and enabling the production of supercritical CO _2_ for the effective operation of CEEGS technologies.

The same scenario as the previous one was considered. After continuous CO _2_ injection for two years (2 million tonnes), as shown in Figure 6b, the bottom hole pressure increases as the injection proceeds and reaches a maximum of 15.5 MPa after two years.

As discussed in 58, this pressure is low and does not allow the production of supercritical CO _2_. The produced CO _2_ reaches the wellhead with a pressure of 5.11 MPa, which is below the supercritical state. Consequently, CO _2_ reaches the wellhead in the gaseous phase. To produce supercritical CO _2_ in this situation, it must either increase the CO _2_ injection rate or extend the duration of the plume stage to more than 2 years, with both possibilities resulting in an increase in the reservoir pressure.

Figure 6c illustrates the distribution of CO _2_ during the plume setup phase, highlighting the amounts trapped, dissolved, and stored in the supercritical phase. A substantial portion of CO _2_ is stored in the supercritical phase, with 12% becoming trapped, and 3.6% dissolving in brine ( Table 11). Notably, the stored CO _2_ in the supercritical phase is essential for heat maintenance in CEEGS technologies.

**Table 11.** Amount of CO _2_ injected, supercritical CO _2_, residual trapped and dissolved in the reservoir during Plume setup phase.

CO _2_ Amount	Moles	Fraction of injected CO _2_
Total injected	4.73873E+10	-
CO _2_ Supercritical (mobile) phase	4.60418E+10	96.4%
CO _2_ Trapped Sg < Sgc / Hysteresis	5.98171E+09	12 %
CO _2_ dissolved in Water	1.71166E+09	3.6%

**Figure 7.**
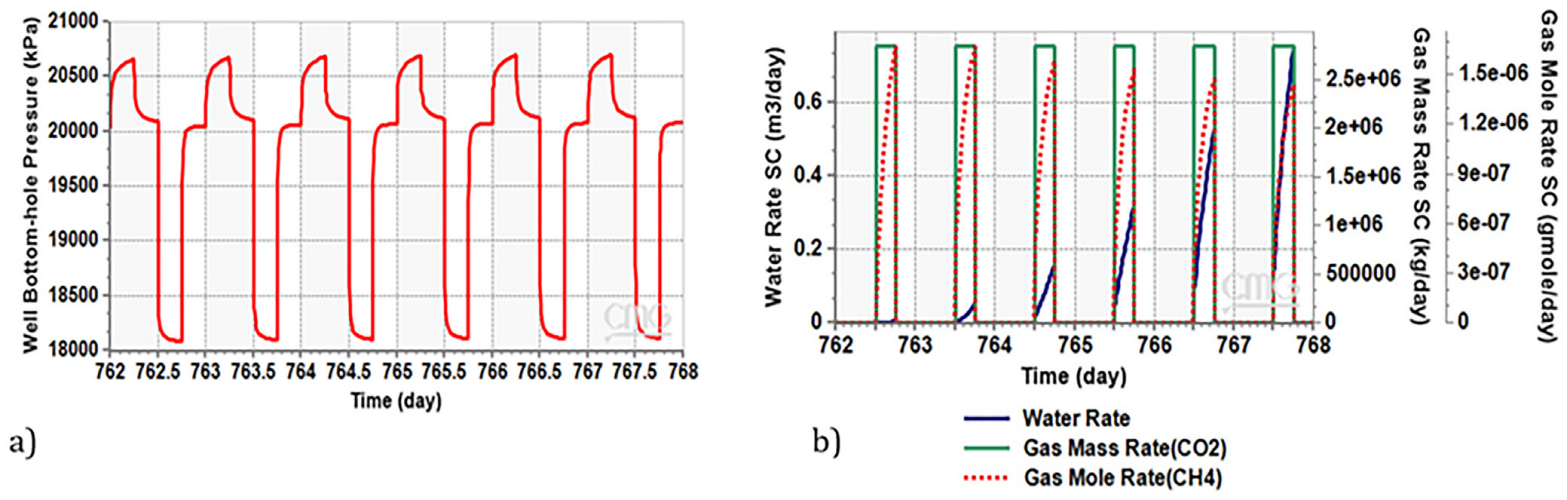
**a**) Bottom Hole Pressure Variation during Charge-Discharge Cycles in Well A Producer, **b**) Water and Methane Dynamics in Production Well During Charge-Discharge Phase.

The variation in Bottom-hole Pressure (BHP) during charge-discharge cycles are illustrated in Figure 7. As evident during the discharge phase (indicated by the low-pressure segment in the curve), Well A is actively producing CO _2_. Notably, the BHP during this phase is 18.1 MPa. According to 58, this pressure level permits the production of supercritical CO _2_, a crucial aspect for the functioning of CEEGS technologies. 

Figure 7b illustrates both the water rate and methane mass rate generated in the production well during the discharge phase. The water volume gradually increasing and reaches 750 litres per day in the last cycle, despite that, this amount remains very low compared to the produced CO _2_ (0.02 %wt). This emphasizes the significance of installing a water separator at the production well, to ensure good functioning of the supercritical CO _2_ surface installation. In contrast, the quantity of methane accompanying the CO _2_ is in the order of milligrams per day. This amount is sufficient for the effective operation of the CO _2_ surface installation.

According to the preliminary results from the hydrodynamic simulation of a generalized depleted gas reservoir, the establishment of a CO _2_ plume is crucial for restoring reservoir pressure to pressure levels compatible with the requirement of the CEEGS and effectively charging the reservoir with CO _2_. This process is essential for establishing a CO _2_ connection between the injection and production wells in the supercritical phase, as outlined by the CEEGS concept. Additionally, it is recommended to reduce the water content in the reservoir to minimize the potential interactions between acidic CO _2_-rich water and the host rock.

## Conclusions

The CO _2_ storage in DHF is of high interest for the proper management of CO _2_ emissions. The storage capacity of DHF and the present infrastructure make them an attractive option for the underground CO _2_ storage. Geochemical interactions between the remaining fluids in the DHF (i.e. water and CH _4_), the mother rock of the reservoir and the injected CO _2_ are critical risks that could cause failure to implement this technology. The quasi steady-state simulations’ software PHREEQC was used to investigate the aquatic geochemical interactions in the present study. Marismas field selected as a model DHF for the CO _2_ injection under supercritical conditions required for the novel CO _2_ based electrothermal energy and geological storage system technology.

The reservoir rock/water/remaining gas (CH _4_) interactions proved that all the main mineralogical phases of the area were in equilibrium in the reservoir except K-feldspar and illite. Gibbsite (Al(OH) _3_), K-mica, and kaolinite were formed as new mineralogical phases after the dissolution of K-feldspar. The reservoir was supersaturated in hematite, goethite, siderite, and K-mica, which caused to glauconite transformation. Talc was also observed as a new phase by the geochemical calculations due to the carbonate rocks alteration. During the CO _2_ injection, it is partially dissolute in the water making the brine more acidic and causing an acid attack to the rock minerals. Carbonate minerals are strongly influenced by this attack while glauconite, talc, and chlorite dissolution were also observed. The prolonged K-feldspar dissolution resumed also after the CO _2_ injection. The liberated ions of these minerals were present in the brine, having the ability to react with each other, resulting in clay minerals formulations. Kaolinite, illite, and Ca-montmorillonite were the main newly formed clay minerals, while only Ca-montmorillonite threatens the permeability of the reservoir due to its swelling properties. In the case of the minimum potential CO _2_ injected concentration, hematite, goethite, and siderite were present in the reservoir. Goethite is a relatively stable phase of Fe-minerals that is not related to pore-clogging. However, during the increase of the CO _2_ concentration, the goethite dissolves and hematite as well as siderite precipitated in a lower extent. As siderite is composed by small colloidal particles, its presence could cause a pore throat clogging effect. Thus, the less siderites percolated in the system, the better.

Summarising, from a geochemical perspective, only Ca-montmorillonite and siderite could cause failures in the investigated reservoir, however, their presence can be easily controlled by anthropogenic changes in the reservoir parameters and especially pH.

This study highlights the technical feasibility of CEEGS (CO _2_-based Electrothermal Energy and Geological storage) technology, emphasizing the geochemical suitability of depleted natural gas reservoirs as underground storage candidates. Depleted gas reservoirs are considered ideal for CO _2_ storage due to their proven structural traps and caprock seals, which effectively prevent the lateral and vertical migration of gas, enabling long-term containment.

For successful CEEGS implementation, simulation results indicate that the establishment of a CO _2_ plume is a critical phase. This plume helps to stabilize or even increase reservoir pressure to levels conducive to CO _2_ storage, reducing the risk of water upconing. Establishing a stable CO _2_ plume between injection and production wells allows for continuous injection and withdrawal rates during the energy storage stage, optimizing the efficiency of the underground part.

Additionally, evaluating the geochemical integrity of depleted gas reservoirs is essential for safe, long-term storage. Key aspects of the storage complex—including the reservoir, caprock, and wells—must be carefully assessed. This assessment is best achieved through an integrated approach, combining field observations, laboratory experiments, and numerical modeling to gain a comprehensive understanding of geochemical stability.

The results of this study suggest that the proposed depleted gas reservoir for the CEEGS technology underground is promising for real-world applications. Future research should focus on exploring the combined effects of reservoir heterogeneity, CO _2_ dissolution, and rate-limited reactions to refine the understanding of this technology's long-term viability.

## Ethics and consent

Ethical approval and consent were not required.

## Data Availability

Zenodo: Scripts for PHREEQC calculations for "CO _2_ sequestration potential in Depleted Hydrocarbon fields – a geochemical approach, DOI: https://doi.org/10.5281/zenodo.14558467 ^84^ *This project contains the following underlying data:* Phreeqc.txt Scripts for PHREEQC calculations Data are available under the terms of the Creative Commons Attribution 4.0 International

## Software availability

PHREEQC, Source code available from: https://www.usgs.gov/software/phreeqc-version-3

CMG-GEM compositional simulator was used with a license provided by the CMG (Computer Modelling Group). An open source tool able to conduct THMC modelling of porous media reservoirs is OPENGEOSYS, with source code available at https://gitlab.opengeosys.org/ogs/ogs.
