# Supplementary material for: CO 2 sequestration potential in Depleted Hydrocarbon fields – A geochemical approach
Source: Open Res Eur. 2025 Apr 4;5:17. Originally published 2025 Jan 20. [Version 2] doi: 10.12688/openreseurope.19280.2 (PMC12166356; doi:10.12688/openreseurope.19280.2)
Supplement: Peer review history

# Reviewer report: Reviewer response for version 2

**DOI:** 10.21956/openreseurope.21775.r52852

**Published:** 2025-06-13

**Authors:** Wendong Wang

**Affiliations:** 1 Key Laboratory of Unconventional Oil & Gas Development (China University of Petroleum (East China)), Qingdao, China

The author has modified the manuscript carefully.

Is the study design appropriate and does the work have academic merit?

Yes

Is the work clearly and accurately presented and does it cite the current literature?

Yes

If applicable, is the statistical analysis and its interpretation appropriate?

Partly

Are all the source data underlying the results available to ensure full reproducibility?

Yes

Are the conclusions drawn adequately supported by the results?

Yes

Are sufficient details of methods and analysis provided to allow replication by others?

Partly

Reviewer Expertise:

CO2 sequestration

I confirm that I have read this submission and believe that I have an appropriate level of expertise to confirm that it is of an acceptable scientific standard.

# Reviewer report: Reviewer response for version 1

**DOI:** 10.21956/openreseurope.20869.r50151

**Published:** 2025-03-03

**Authors:** Olabode Olakoyejo

**Affiliations:** 1 University of Lagos, Lagos, Nigeria

**Comments of the Reviewer:**

**Paper Title**:  CO2 sequestration potential in Depleted Hydrocarbon fields – A geochemical approach

**General Comments**

 The manuscripts presented a numerical investigation of the potential CO2 capturing and storage in depleted hydrocarbon fields (DHF) using PHREEQC software. The Marismas 3 field was used as a model for the analysis of CO2 injection and interactions with a carbonate-silisiclastic reservoir under supercritical conditions based electrothermal energy and geological storage system technology (CEEGS).

 The research is of interest and the manuscript report relevant and useful in the CO2 capturing and storage in depleted hydrocarbon fields (DHF)

 The paper is, in general, well-written and duly illustrated. The title of the paper depicts the nature and scope of the study

 The appropriate keywords were selected

 The table and the figures are well analysed and could easily be interpreted by the reader.

 The referencing citing and listing styles are consistent and okay

**Specific comments**

**Abstract**

 The Abstract is well detailed and sufficiently described the study subject matter. The objectives of the article are very clear. However, the result and conclusion parts of the abstract is not robust.

**INTRODUCTION**

****

 The introduction is detailed and satisfactory. The authors shown some level of novelty of the research work to demonstrate their contribution to the body of knowledge. The authors cited and listed current and relevant references.

  Roquero *et al*., 2013 is not properly cited. It should follow the right style of citing as  others. Also was not listed in the reference section

**METHODOLOGY**

****

 The study area and Reservoir features with Figure 1 and 2 well detailed for the problem description. However, Equations [4] – [7] not well written mathematically. It is advised to write the equations in terms of variable and Greek letters. For example, RHO could written as ρ

 Equations [8] may not necessary be equation but boundary condition with lower limit of M _CO2_ = 367,290,525 kg and upper limit = 1,836,452,625 kg

 This could be written as

 MCO 2 ≤ 367,290,525 kg ≤ 1,836,452,625 kg  [8]

**PHREEQC** **and** **CMG-GEM numerical modelling tool** **s:**

 This subsection is well written. However, the authors did not write the governing equations that describe the behaviour of fluid flow of CO _2_ injection in the DHF problem

 There is need for grid independence test (See Figure 4) to ensure accuracy of the model

 Also. there is need for the authors to validate the current work/model with that of related work ( either numerical/empirical or experimentation) in the literature to show that their model and code are okay and working

 In page 11 column 1 line 4; “van ‘t Hoff equation’” should be cited and referenced

**RESULT AND DISCUSSION**

**C** **MG-GEM calculations**

 Detailed and okay

 The results are well detailed and properly and scientifically discussed. The table and the figures are well analysed

**Conclusion:**

 The conclusion is robust enough and addressed the subject matter

**REFERENCES**

 The references were satisfactory with relevant and latest references Cited references were listed. However. The Authors need to be consistence with the style of references for example page 6 paragraph 2, line 9  “Roquero *et al*., 2013” does not follow the style of reference and was not list in the reference section.

**Recommendation**

 The result in general is thorough and robust to describe the solution to the problem. The paper should be accepted after necessary corrections by the authors as indicated above.

****

**Thank you sir for the opportunity.**

Is the study design appropriate and does the work have academic merit?

Yes

Is the work clearly and accurately presented and does it cite the current literature?

Yes

If applicable, is the statistical analysis and its interpretation appropriate?

Yes

Are all the source data underlying the results available to ensure full reproducibility?

Yes

Are the conclusions drawn adequately supported by the results?

Yes

Are sufficient details of methods and analysis provided to allow replication by others?

Yes

Reviewer Expertise:

Dr. Olakoyejo has expertise in Computational thermofluids, with a specialised focus on thermal management in heat-generating devices at macro, micro, and nanoscales. His work leverages Constructal Theory, Computational Fluid Dynamics (CFD), and Artificial Intelligence (AI)-driven techniques to optimize energy systems critical to modern technological advancements.Dr. Olakoyejo is also at the forefront of two-phase flow boiling research, using numerical modeling (CFD) to study phase-change phenomena in heat exchangers, microchannels, and industrial cooling systems. His simulations provide insights into bubble dynamics, heat flux distribution, and flow instabilities, aiding in the design of more efficient thermal management systems for energy and electronics applications.His research extends to wind where he integrates advanced condition monitoring and predictive models using data analysis and machine learning in wind turbine to detect potential failure of wind turbine before it happens to ensure maximize energy output, reliability, and efficiency, and reduce operational costs of the wind turbineAlso, he is in carbon sequestration modeling to address air pollution challenges associated with industrial emissions and gas flaring integrating condition monitoring and predictive models. Using AI-driven predictive models and CFD simulations, he evaluates carbon capture techniques, optimizing sequestration processes for enhanced environmental sustainability, enhance system reliability, maximize energy output

I confirm that I have read this submission and believe that I have an appropriate level of expertise to confirm that it is of an acceptable scientific standard.

# Response

**Published:** 2025-03-31

**Authors:** Ελένη Γιαννή

**Affiliations:** Department of Environment, Ionian University, Zante, Ionio, Greece

We would like to thank the reviewer for his comments. Please see below are detailed responses.  The manuscripts presented a numerical investigation of the potential CO _2_ capturing and storage in depleted hydrocarbon fields (DHF) using PHREEQC software. The Marismas 3 field was used as a model for the analysis of CO _2_ injection and interactions with a carbonate-silisiclastic reservoir under supercritical conditions based electrothermal energy and geological storage system technology (CEEGS).

 The research is of interest and the manuscript report relevant and useful in the CO _2 _capturing and storage in depleted hydrocarbon fields (DHF)

 The paper is, in general, well-written and duly illustrated. The title of the paper depicts the nature and scope of the study

 The appropriate keywords were selected

 The table and the figures are well analysed and could easily be interpreted by the reader.

 The referencing citing and listing styles are consistent and okay

   Response: We would like to thank the reviewer for his kind comments. 

**Specific comments**

**Abstract**

 The Abstract is well detailed and sufficiently described the study subject matter. The objectives of the article are very clear. However, the result and conclusion parts of the abstract is not robust.

 Response: Thank you for your comment. We tried to modify the conclusion part of the abstract to be more robust, however the limitation of abstract words does not allow us to furtherly improve it. 

**INTRODUCTION**

****

 The introduction is detailed and satisfactory. The authors shown some level of novelty of the research work to demonstrate their contribution to the body of knowledge. The authors cited and listed current and relevant references.

  Roquero *et al*., 2013 is not properly cited. It should follow the right style of citing as  others. Also was not listed in the reference section

 Response: You are right. The reference was added similarly to the others, while it was also added in the reference section.

**METHODOLOGY**

****

 The study area and Reservoir features with Figure 1 and 2 well detailed for the problem description. However, Equations [4] – [7] not well written mathematically. It is advised to write the equations in terms of variable and Greek letters. For example, RHO could written as ρ

 Equations [8] may not necessary be equation but boundary condition with lower limit of M _CO2_ = 367,290,525 kg and upper limit = 1,836,452,625 kg

 This could be written as

 MCO 2 ≤ 367,290,525 kg ≤ 1,836,452,625 kg  [8]

 Response: Thank you for your valuable comments. The equations corrected accordingly.

**PHREEQC and CMG-GEM numerical modelling tools:**

 This subsection is well written. However, the authors did not write the governing equations that describe the behaviour of fluid flow of CO _2_ injection in the DHF problem

 There is need for grid independence test (See Figure 4) to ensure accuracy of the model

 Also. there is need for the authors to validate the current work/model with that of related work ( either numerical/empirical or experimentation) in the literature to show that their model and code are okay and working

 In page 11 column 1 line 4; “van ‘t Hoff equation’” should be cited and referenced Response: Thank you for your comment. Figure 4 was replaced to ensure accuracy of the model, and the citation of the equation was added within the manuscript.

**RESULT AND DISCUSSION**

**CMG-GEM calculations**

 Detailed and okay

 The results are well detailed and properly and scientifically discussed. The table and the figures are well analysed Response: Thank you. 

**Conclusion:**

 The conclusion is robust enough and addressed the subject matter

  Response: Thank you. 

**REFERENCES**

 The references were satisfactory with relevant and latest references Cited references were listed. However. The Authors need to be consistence with the style of references for example page 6 paragraph 2, line 9  “Roquero *et al*., 2013” does not follow the style of reference and was not list in the reference section.

 Response: You are right. The reference was added similarly to the others, while it was also added in the reference section. **Recommendation**

 The result in general is thorough and robust to describe the solution to the problem. The paper should be accepted after necessary corrections by the authors as indicated above.

 Response: Thank you. 

# Reviewer report: Reviewer response for version 1

**DOI:** 10.21956/openreseurope.20869.r50150

**Published:** 2025-02-05

**Authors:** Wendong Wang

**Affiliations:** 1 Key Laboratory of Unconventional Oil & Gas Development (China University of Petroleum (East China)), Qingdao, China

1. While both PHREEQC and CMG-GEM are established software, the paper doesn't present any validation of the model parameters against experimental or field data, which raises questions about the reliability of the predictions.

 2. The study makes significant assumptions about the mineralogical composition of the reservoir, particularly in cases where specific data wasn't available. How these assumptions might impact the overall conclusions？

 3. While the paper focuses on geochemical interactions, the potential geomechanical impacts of mineral dissolution and precipitation on reservoir integrity are only briefly mentioned.

 4. While some references are made to literature, a systematic comparison would have helped contextualize the findings.

 5. While the paper discusses various reactions, the time scales over which these reactions occur and their implications for long-term storage are not adequately explored.

 6. The paper's figures and tables could be more informative. Some figures lack proper labeling and scale bars, while tables contain raw data without adequate explanation of their significance in the context of the study's objectives.

Is the study design appropriate and does the work have academic merit?

Yes

Is the work clearly and accurately presented and does it cite the current literature?

Yes

If applicable, is the statistical analysis and its interpretation appropriate?

Partly

Are all the source data underlying the results available to ensure full reproducibility?

Yes

Are the conclusions drawn adequately supported by the results?

Yes

Are sufficient details of methods and analysis provided to allow replication by others?

Partly

Reviewer Expertise:

CO2 sequestration

I confirm that I have read this submission and believe that I have an appropriate level of expertise to confirm that it is of an acceptable scientific standard, however I have significant reservations, as outlined above.

# Response

**Published:** 2025-03-31

**Authors:** Ελένη Γιαννή

**Affiliations:** Department of Environment, Ionian University, Zante, Ionio, Greece

We would like to thank the reviewer for his comments. Please see our detailed responses below. 

1. While both PHREEQC and CMG-GEM are established software, the paper doesn't present any validation of the model parameters against experimental or field data, which raises questions about the reliability of the predictions.

**Response: **We would like to thank the reviewer for his comment. We agree with the reviewer that there are no experimental data that can confirm the modelled geochemical approach, however the study is a first attempt to conceptualize the area pending further research and data availability. Additionally, this first conceptualization is based on data derived from traditional geological mapping, concerning mineralogical phases that are not frequently meet in similar carbonate-silisiclastic reservoirs but are present in the study area, such as glauconite, and on confidential data from Marismas field related to the remaining water characteristics in the reservoir and porosity of the reservoirs. A relative sentence was added in the introduction part for the reader's better understanding.

1. The study makes significant assumptions about the mineralogical composition of the reservoir, particularly in cases where specific data wasn't available. How these assumptions might impact the overall conclusions？

**Response: **Based on the available literature and specifically available geological maps of the area, stratigraphic columns and X-Ray diffraction analysis of samples taken in the area and keeping into consideration the XRD detection limits, we believe that all the main mineralogical phases are taken into consideration. Thus, we believe that our calculations represent the main geochemical interactions that could take place in Marismas fields. However, as as the heterogeneity of rock microstructure is possible even in micro-scale environments, we do not exclude the existence of additional geochemical reactions caused by the action of impurities or by the dominance of other mineralogical phases than them that was taken into consideration. A relative sentence was included within the text for the sake of completeness.

1. While the paper focuses on geochemical interactions, the potential geomechanical impacts of mineral dissolution and precipitation on reservoir integrity are only briefly mentioned.

**Response:** Thank you for your comment. Additionally, literature was added in the manuscript to robust the geomechanical impacts of mineral dissolution and precipitation on reservoir integrity (see within the text – mainly scenario 1 and 2).

1. While some references are made to literature, a systematic comparison would have helped contextualize the findings.

**Response:** Thank you for your comment. As the PHREEQC software is not widely used in the area of CO _2_ underground injection in depleted hydrocarbon fields, its use in several types of reservoirs from a mineralogical point of view is of high interest for our research group which is willing to work in future research in a systematic comparison among them.

1. While the paper discusses various reactions, the time scales over which these reactions occur and their implications for long-term storage are not adequately explored.

**Response:** Thank you for your comment. In case of CMG-GEM modelling the time used for the calculations is for a two years CO _2_ injection duration (referred in section “CMG-GEM model description and input values”). In case of PHREEQC calculations, there are no time-specific calculations as the geochemical calculations are a matter of reaching the chemical equilibrium of the system without referring to the time needed for this phenomenon. As this was not clearly given in the manuscript, a relative sentence was added in the “PHREEQC scenarios and input values” section.  

1. The paper's figures and tables could be more informative. Some figures lack proper labeling and scale bars, while tables contain raw data without adequate explanation of their significance in the context of the study's objectives.

**Response:** Indeed, some of the figures were not well prepared. Thus, Figures 4-7 were replaced. It is true that tables contain raw data, however in each Table it is referred that “Throughout the report the number of moles and other chemical quantities are provided in this format so that accuracy is not lost if attempting reproducibility of the results.” The purpose of using raw data is the area representation with a high accuracy when creating the input files for both the selected software.
